# Impact of Noncompensating Ions on the Electrochemical Performance of n-Type Polymeric Mixed Conductors

**DOI:** 10.1021/jacs.4c17579

**Published:** 2025-04-07

**Authors:** David Ohayon, Amer Hamidi-Sakr, Jokubas Surgailis, Shofarul Wustoni, Busra Dereli, Nimer Wehbe, Stefan Nastase, Xingxing Chen, Iain McCulloch, Luigi Cavallo, Sahika Inal

**Affiliations:** † Organic Bioelectronics Laboratory, Biological and Environmental Sciences and Engineering Division, King Abdullah University of Science and Technology (KAUST), Thuwal 23955-6900, Saudi Arabia; ‡ Institute for Functional Intelligent Materials, National University of Singapore, Singapore 117544, Singapore; § Departments of Chemistry and Chemical & Biomolecular Engineering, National University of Singapore, Singapore 119077, Singapore; ∥ Catalysis Research Center, Physical Sciences and Engineering Division, KAUST, Thuwal 23955-6900, Saudi Arabia; ⊥ Imaging and Characterization Core Lab, King Abdullah University of Science and Technology (KAUST), Thuwal 23955, Saudi Arabia; # KAUST Solar Center, Physical Sciences and Engineering Division, KAUST, Thuwal 23955-6900, Saudi Arabia; ∇ Department of Chemistry, University of Oxford, Oxford OX1 3TF, United Kingdom

## Abstract

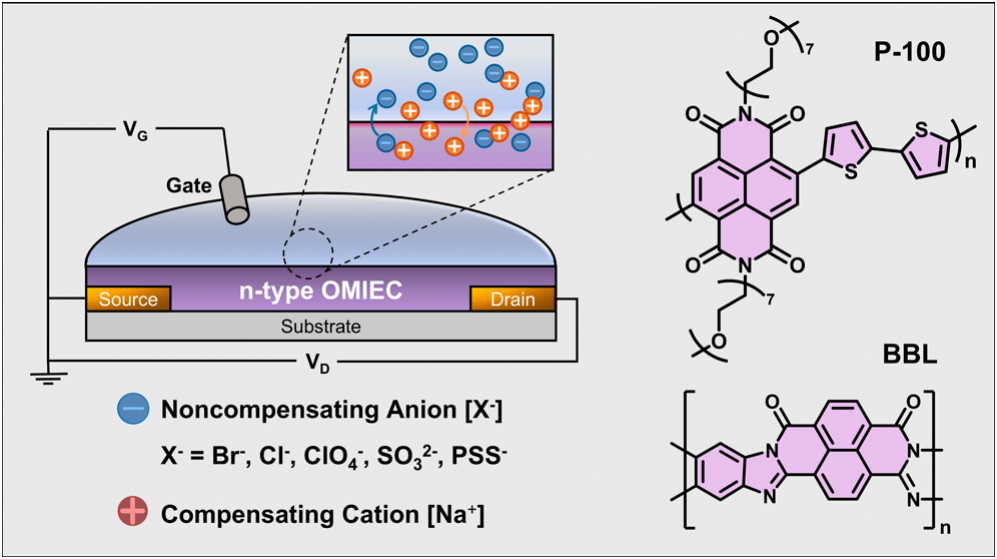

Organic mixed ionic-electronic conductors (OMIECs) have emerged as essential materials for applications in bioelectronics, neuromorphics, and energy storage, owing to their ability to transport both ions and electrons. While significant progress has been made in understanding their operation, the role of noncompensating ions in polymer redox processes remains underexplored, particularly in the context of their impact on charge compensation and device performance. In this study, we systematically investigate the influence of noncompensating ions on the performance of n-type OMIECs with and without polar side chains, focusing on their interactions with electrolytes containing anions from the Hofmeister series. Our findings reveal a stark contrast in charging behavior and organic electrochemical transistor (OECT) performance based on side-chain chemistry. Polar oligoether side chains promote interactions with anions, resulting in significant performance variations. We demonstrate the critical role of polymer side-chain interactions with the different anions, where polyatomic anions capable of infiltrating the film degrade device performance, particularly in terms of transconductance and operational stability. In contrast, OMIECs without side chains exhibit performance independent of the noncompensating ion nature. Through electrochemical analysis, spectroscopic techniques, and molecular dynamics simulations, we provide a comprehensive understanding of how ion incorporation and polymer–electrolyte interactions shape device behavior. This study highlights the transformative role of side-chain functionality in tailoring the properties of the OMIEC and offers a design framework for high-performance OECTs, enabling advancements in biosensing, neuromorphic computing, and beyond.

## Introduction

Organic electrochemical transistors (OECTs) are amplifying transducers that operate in aqueous electrolytes at low voltages (<1 V).^1^ These features, combined with the nontoxic nature of channel materials, make OECTs uniquely suited for direct interfacing with biological systems.^2^ OECTs have found a wide range of potential applications, including neuromorphic devices,^3,4^ and sensors that quantify concentrations of ions,^5^ proteins,^6^ and metabolites,^7^ or monitor the integrity of cell membranes^8^ and neural activity.^9^ In the OECT architecture, the channel, often comprising an ion-permeable conjugated polymer, known as an organic mixed ionic-electronic conductor (OMIEC), is exposed to an electrolyte with an immersed gate electrode. When a gate voltage (*V*_G_) is applied, electrolyte ions that have the same charge with the polarization of the gate electrode migrate into the channel and compensate for the electronic charges injected into the film through the source electrode. For example, for a channel made of an undoped n-type (electron transporting) OMIEC, a positive *V*_G_ drives the compensating ions (i.e., cations) into the film, stabilizing the electrons and thereby increasing the channel current (*I*_D_). The extent to which the OMIEC film couples these ions with electronic charges and the speed of the electronic charge transport determine the signal amplification in the OECTs, which is measured as the device’s transconductance (*g*_m_ = ∂*I*_D_/∂*V*_G_). The *g*_m_, at saturation, is described by the following equation:
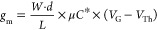
1where *L*, *W*, and *d* are the channel dimensions: length, width, and thickness, respectively, and (*V*_G_ – *V*_Th_) is the overdrive voltage with a threshold voltage *V*_Th_, being the minimum *V*_G_ required to initiate a conductive path. The *g*_m_ is affected by two key properties of the OMIEC: electronic mobility (μ) and volumetric capacitance (C*).^10^ These properties can be tuned by modifying the chemical structures of the OMIECs. For instance, polar ethylene glycol (EG) side chains have been anchored onto hydrophobic conjugated backbones to promote hydrated ion uptake and transport within the film.^11^ Adjusting the OMIEC’s chemical composition, such as the ratios of polar side chains to aliphatic ones or the positioning of polar side chains with respect to the backbone, can impact *V*_Th_ and *g*_m_.^12−14^ These OECT properties can also be modulated by the type of gate electrode used (polarizable vs nonpolarizable). Moreover, studies have shown how the type of compensating ions of the electrolyte, those that balance electronic charges in the channel, influences OECT performance.^15,16^ For example, Cendra et al. investigated the effect of different sodium (Na^+^)-based aqueous electrolytes on hole-transporting (p-type) OECT characteristics, finding that larger and less hydrated anions (such as SbF_6_^–^) resulted in OECTs with higher *g*_m_ than smaller anions (like Cl^–^).^17^ Similarly, Flagg et al. demonstrated that molecular anions (such as TFSI^–^ or PF_6_^–^) led to higher channel currents and lower *V*_Th_ in p-type OECTs compared to smaller atomic anions (such as Cl^–^).^18,19^ In addition to the role of compensating ions, the water transported into the channel during operation must be considered, as the hydration level of dopant ions influences the morphology of the film, thereby charge transport.^20,21^ While these studies suggest the importance of electrolyte choice on device performance, no clear guidelines on the optimal pairing of the electrolyte and the OMIEC exist, suggesting a significant gap in understanding how these devices actually operate and how polymer films interact with electrolyte ions.

In addition to compensating ions and water, aqueous electrolytes contain noncompensating ions with opposite charge. These are ions that do not participate in stabilizing the electronic charges in the OMIECs. Therefore, they are viewed as “inactive bystanders”, and their possible role in the operation of the OECT has been largely overlooked. However, a few recent studies found evidence that noncompensating ions could influence the electrochemical charging performance of the OMIECs. Flagg et al. showed that these ions could contribute to charge neutrality during p-type electrochemical doping, affecting the doping onset and efficiency.^22^ Lutkenhaus et al. demonstrated that the choice of electrolyte, particularly the chaotropic or kosmotropic character (i.e., the tendency to strengthen or disrupt the hydrogen bonding network of water), symmetry, and size of the ions, influenced aqueous battery performance for nonconjugated radical polymers.^23^ They found that, depending on the chao-/kosmotropic character of both ions and the experiment’s duration, polymer oxidation could occur either via anion uptake or cation expulsion. These findings suggest that noncompensating anions may not be as passive as once thought, opening up a new material-electrolyte design matrix for device performance optimization.

**Figure 1:**
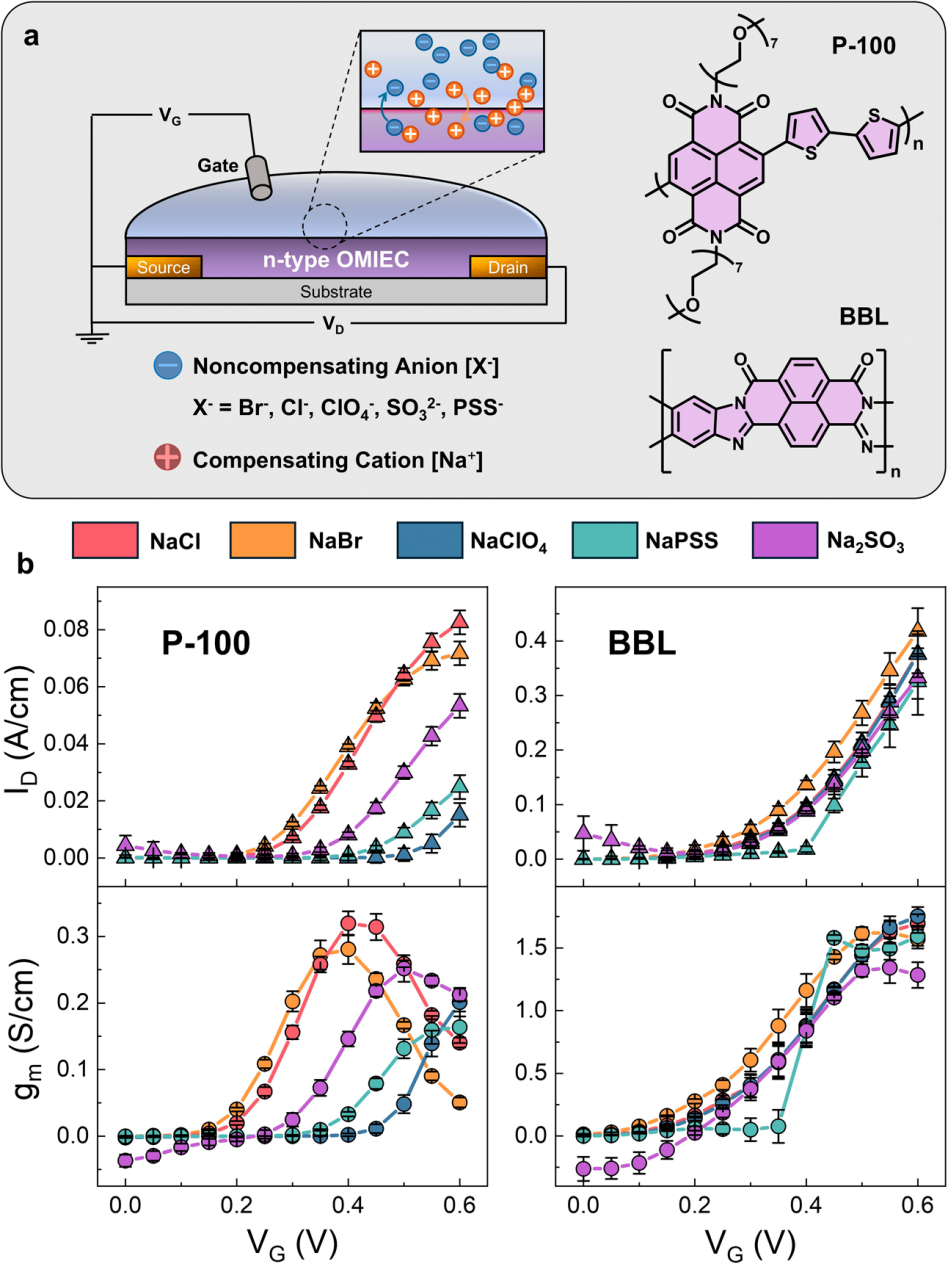
(a) Chemical structure of P-100 and BBL, with the schematic of the OECT and the five sodium-based electrolytes. (b) Transfer curves obtained at *V*_D_ = 0.6 V and the corresponding thickness-normalized *g*_m_ as a function of *V*_G_ for n-type OECTs operated in the different electrolytes. Error bars represent the standard variations of at least 6 different devices. The gate electrode was a Ag/AgCl pseudo-reference electrode with the same potential in each electrolyte. Na^+^ concentration was 0.1 M in all electrolytes.

In this study, we investigate the impact of the noncompensating anions on n-type OECT performance. We choose to work with n-type OMIECs as they remain underexplored relative to their p-type counterparts despite their potential in emerging applications such as enzymatic biosensors, complementary circuits, capacitors, and biofuel cells.^24^ Specifically, we investigate how the size, valency, atomicity, surface charge density, and chaotropic/kosmotropic character of these anions impact the electrochemical behavior of selected n-type OMIECs using a combination of electrochemical techniques with physicochemical ones such as X-ray photoelectron spectroscopy (XPS), dynamic secondary ion mass spectrometry (SIMS), and quartz crystal microbalance with dissipation monitoring (QCM-D). Our main channel material is a donor–acceptor copolymer with a naphthalene-1,4,5,8-tetracarboxylic-diimide-bithiophene (NDI-T2) backbone functionalized with EG side chains, referred to as P-100 (Figure 1a). To assess the role of side chains, we compare P-100 with a side-chain-free polymer, poly(benzimidazobenzophenanthroline) (BBL). Our findings reveal distinct ion infiltration profiles for the NDI-T2 film depending on the anion type, with monatomic anions yielding better device stability, higher *g*_m_, and lower *V*_Th_ than polyatomic anions. Passive infiltration of noncompensating anions generally delayed the device’s onset voltage, likely due to the energetic cost associated with anion expulsion from the polymer bulk. These results were interpreted through molecular dynamics (MD) simulations and density functional theory (DFT) calculations, which provided insights into how ion-polymer interaction energies influence device performance. The transport mechanism of ions was explored using the Donnan steric pore model with dielectric effect (DSPM-DE), which suggested that OMIECs can be conceptualized as nanofiltration (NF) membranes to predict ion-polymer interactions. Overall, our study reveals the overlooked role of noncompensating ions in n-type OECT operation and highlights the rational selection of OMIEC/electrolyte combinations for optimizing electrochemical device performance and enhancing their functionality.

## Results and Discussion

### OECT Characteristics

**Figure 2:**
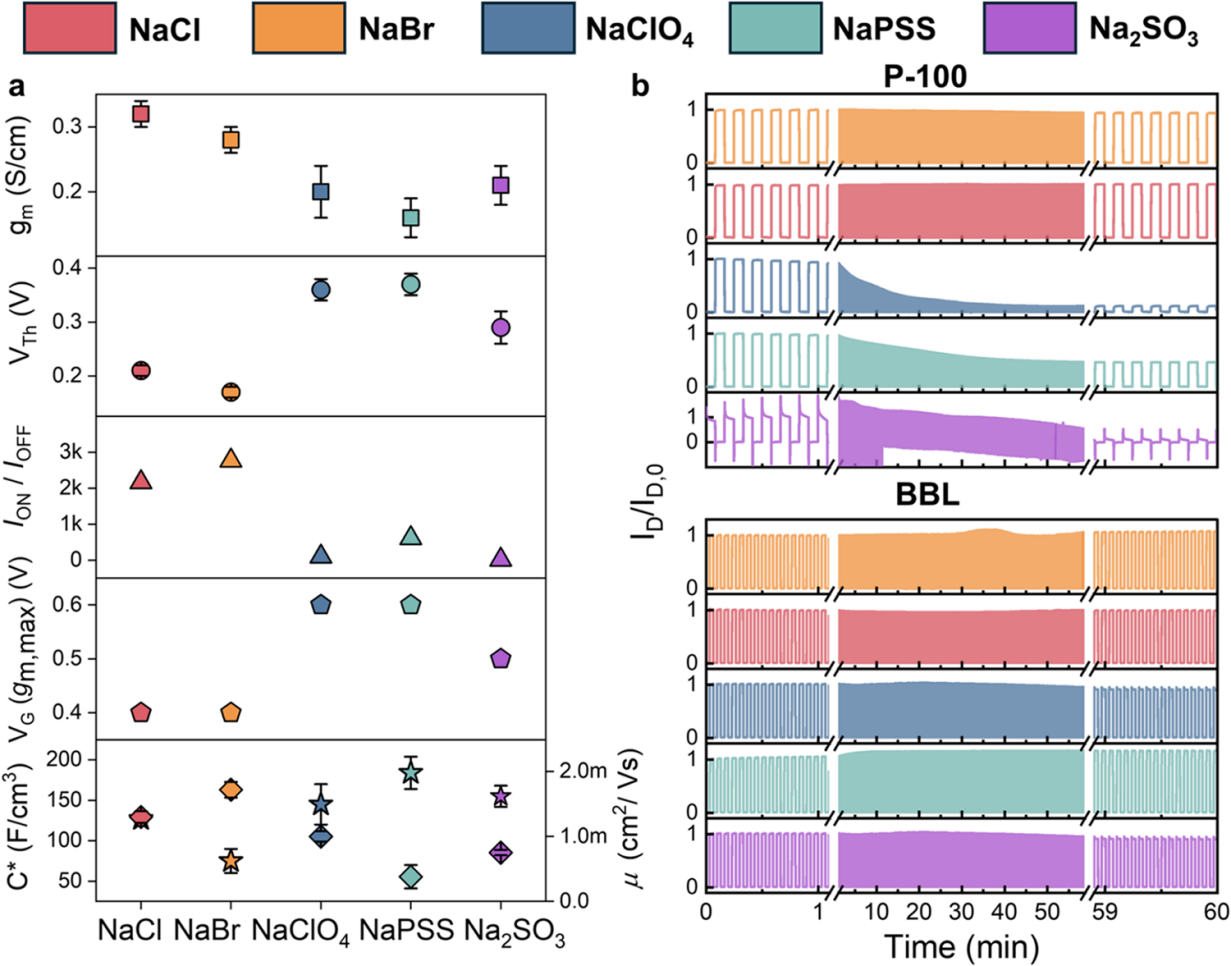
(a) P-100 OECT steady-state metrics showing from the top row down the normalized *g*_m_, the threshold voltage *V*_Th_, the *I*_ON_/*I*_OFF_, the *V*_G_ of the *g*_m,max_, volumetric capacitance C* (stars), and the electron mobility μ (diamonds). Error bars represent the standard deviations of at least 6 different devices. (b) Operational stability measurements for the P-100 and BBL OECTs operating in the five electrolytes. Drain currents (*I*_D_) were normalized to the initial drain current of the first pulse (*I*_D,0_). The devices were operated at *V*_D_ = 0.5 V and switched between *V*_G_ = 0.5 V (ON) and *V*_G_ = −0.1 V (OFF) at 10 s intervals. The gate electrode was an Ag/AgCl pseudo-reference electrode.

We investigated the effect of noncompensating anions on n-type OECT performance by fabricating OMIEC-based channels with dimensions of 100 μm width and 10 μm length and gating them using an Ag/AgCl electrode with a stable potential in Na^+^-based electrolytes at a concentration of 0.1 M. We used five electrolytes with Na^+^ as the compensating cation and different anions based on the Hofmeister series: chloride (Cl^–^), bromide (Br^–^), and perchlorate (ClO_4_^–^) as chaotropes; sulfite (SO_3_^2–^) and poly(styrenesulfonate) (PSS^–^) as kosmotropes (see Supporting Information, Discussion 1 on the Hofmeister series). Steady-state measurements revealed significant differences in the performance of P-100 devices depending on the electrolyte used (Figures 1b and S1). Notably, while P-100 devices showed considerable variations in current output based on the electrolyte, BBL devices exhibited minimal sensitivity to electrolyte identity. Specifically, P-100 OECTs operated with monatomic anions (X = Br^–^ or Cl^–^) displayed a significantly lower threshold voltage *V*_Th_ (*V*_Th_ ≤ 0.21 V) and higher *I*_ON_/*I*_OFF_ ratios (10^4^), achieved an average peak *g*_m_ ≥ 0.28 S/cm, and reached their maximum *g*_m_ (*g*_m,max_) at lower gate potentials (*V*_G_ ≤ 0.4 V) compared to those with polyatomic anions (X = ClO_4_^–^ or SO_3_^2–^) (Figure 2a).

As reported by Cendra et al., the solution pH can affect the behavior of p-type OECTs.^17^ To investigate whether pH variations could explain the observed trends in P-100 OECT performance, we measured the pH of the five electrolytes, which ranged from 5 (for NaPSS) to 9.8 (for Na_2_SO_3_). To ensure that the observed differences in P-100 OECT performance were not driven by changes in solution pH, we recorded the device characteristics in NaCl solutions with pH values adjusted between 3 and 10. Our results show that the electrolyte pH had no impact on either *V*_Th_ or the *g*_m_ (Figure S2), confirming that the performance differences are attributable solely to the nature of the noncompensating ions.

The properties of the OMIECs also vary depending on the electrolyte used. We performed electrochemical impedance spectroscopy (EIS) measurements at an offset voltage corresponding to the respective *V*_G_ applied to reach the *g*_m,max_ in the various electrolytes (Figure S3). From the EIS data, we calculated C* values.^25^ When the P-100 film is doped in the electrolyte with monatomic anions, the C* values are lower than those with polyatomic anions (Figure 2a). For instance, films doped with NaCl at 0.4 V vs Ag/AgCl exhibited a C* of 127 ± 8 F/cm^3^, while films doped with NaPSS at 0.6 V vs Ag/AgCl had a C* of 184 ± 20 F/cm^3^. The electronic mobility values were extracted by substituting the relevant parameters in eq 1. P-100 devices had the highest mobility with the monatomic ions with μ = 1.72 ± 0.12 × 10^–3^ cm^2^/(V s) for X = Cl^–^ and μ = 1.32 ± 0.9 × 10^–3^ cm^2^/(V s) for X = Br^–^ (Figure 2a).

Next, we investigated the operational stability of the n-type OECTs in different electrolytes (Figure 2b). Devices were switched “on” and “off” for 360 cycles for 1 h. While BBL devices exhibited consistent stability regardless of the anion type, P-100 devices with monatomic anions demonstrated better stability than those with polyatomic anions, retaining nearly 100% of the initial performance. The devices in NaClO_4_ suffered performance losses of up to 90%. Overall, the type of anion used in n-type OECTs based on glycolated P-100 had a substantial impact on the devices’ performance. P-100 devices performed notably better with monatomic anions, whereas those using polyatomic anions deteriorated more rapidly. On the other hand, BBL devices maintained durability and performance regardless of the anion type. These discrepancies suggest that noncompensating anions play a critical role in the operation of glycolated OMIEC-based OECTs but have a minimal effect on side-chain-free OMIEC-based ones. Importantly, the superior performance observed with monatomic anions remained consistent when the gate electrode was switched to Pt (Figure S4 and Supporting Information, Discussion 2).

### Ion Infiltration and Elemental Analysis

To establish a fundamental understanding of ion-OMIEC interactions, we apply the concept of nanofiltration (NF), commonly used to describe membrane selectivity toward different electrolytes.^26,27^ In NF membrane systems, membrane filtration efficiency, defined as the membrane’s ability to separate solutes from the electrolyte, is determined by the membrane’s charged groups, polarity, and the behavior of electrolyte ions at the electrolyte-membrane interface.^26,28^ Polar polymeric NF membranes, such as polyamide, poly(ether sulfone), and sulfonated polysulfone, are widely used in NF applications.^28,29^ The flux of solutes (i.e., ions) through the membrane is governed by the extended Nernst–Planck equation, which accounts for diffusion (movement of ions down a concentration gradient), convection (transport of ions through bulk fluid flow), and electromigration (movement of ions driven by a potential gradient across the membrane).^26^ Depending on the membrane exclusion properties (such as the pore size and charged groups), certain solutes can be excluded from infiltrating the film. Extensive studies on NF describe three exclusion mechanisms, which are captured by the Donnan steric pore model with dielectric effects (DSPM-DE) model: steric hindrance, Donnan equilibrium, and dielectric exclusion.^26,30^ Dielectric exclusion, in particular, refers to the energy barrier caused by the ion’s hydration shell shedding, which occurs due to the difference in dielectric constants between the aqueous solution and the membrane.

**Figure 3:**
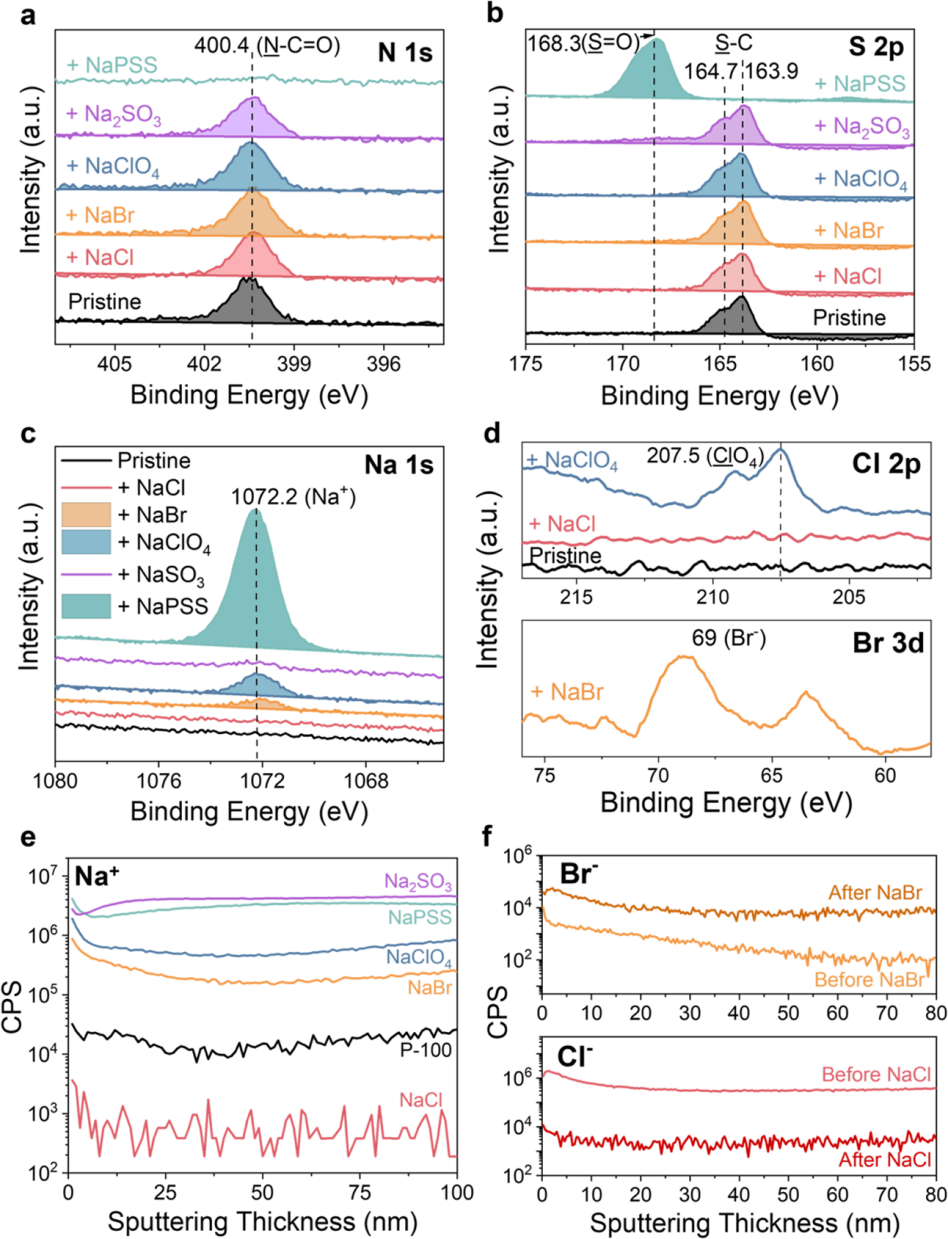
High-resolution XPS and SIMS depth profiling curves show the elemental analysis of the major constituents of the P-100 films after exposure to various electrolytes. (a) N 1s signal displaying the N–C=O contribution (binding energy = 400.4 eV); (b) S 2p signal showing the S–C contribution (binding energy = 164.7 eV (S 2p_1/2_) and 163.9 eV (S 2p_3/2_)); (c) Na 1s signal (binding energy = 1072.2 eV); (d) Cl (2p) signal (binding energy = 207.5 eV) and Br (3d) signal (binding energy = 69 eV). (e) Na^+^ SIMS curves. The Na^+^ signal has a higher intensity when the films are exposed to all electrolytes, except for the film exposed to NaCl compared to the pristine film. The signal of Na^+^ in the pristine film originates from ambient contamination. (f) The higher Br 3d signal after exposure to NaBr suggests that Br^–^ infiltrates the film (top). The lower levels of Cl^–^ after exposure to NaCl suggest that Cl^–^ is excluded from the films (bottom).

P-100 films are polar due to EG side chains and we found that their surface is negatively charged in common buffers with a ζ-potential of −28.87 ± 4.3 mV.^31^ It is also known that OMIECs like P-100 swell passively when exposed to an aqueous electrolyte.^32,33^ Due to the surface charge and swollen network of the P-100 film, we hypothesize that the ion transport and exclusion concepts observed in NF membranes can be extended to our OMIEC/electrolyte system. For instance, the ionic concentration gradient at the electrolyte/polymer interface drives the transport of water molecules into the polymer bulk, leading to swelling of the membrane and the generation of osmotic pressure.^21,34^ The degree of swelling then depends on the osmotic pressure exerted by ion concentration differences and the film’s affinity for water. To evaluate this hypothesis, we performed elemental analysis on films hydrated in different electrolytes. We used XPS to examine the surface of P-100 and coupled it to SIMS measurements to identify the elemental distribution within its bulk. These measurements could allow us to determine whether ions diffused throughout the film (detectable within the XPS penetration depth) or were excluded from the polymer. XPS analysis reveals the characteristic N 1s (400.4 eV) and S 2p (164 eV) signals of P-100, corresponding to the N–C=O and S–C bonds of the polymer backbone, respectively (Figure 3a,b). These signals were present for almost all electrolytes without any noticeable shifts, indicating that P-100 retains its molecular structure after electrolyte exposure. Only in NaPSS, the N–C=O and S–C signals were suppressed, with a new signal appearing at 168.3 eV, corresponding to the S=O bond of the PSS anion.^35^ This result suggests that PSS adsorbs onto the film surface, forming a layer at least 10 nm thick (i.e., the XPS penetration depth). We propose that PSS^–^ chains are excluded from entering the film due to steric hindrance, consistent with the DSPM-DE model.

While the film remains intact after electrolyte exposure, we find traces of some salts on its surface. Figure 3c shows the presence of Na^+^ at 1072.2 eV^41^ in all films *except* for the one exposed to NaCl. The Na^+^ signal is the strongest in the film exposed to NaPSS, followed by that of NaClO_4_ and NaBr. A zoomed-in spectrum reveals a Na^+^ peak for Na_2_SO_3_ as well (Figure S5). In addition to Na^+^, the films passively took up some anions. XPS data in Figure 3d indicates the presence of Br^–^ and Cl^–^ (from ClO_4_), detected at around 69 and 207.5 eV, respectively. The S 2p spectrum of the film, which has been immersed in Na_2_SO_3_, exhibits signals for the S=O bond from the SO_3_^2–^ anion and the S–C bond of the polymer backbone (Figure S5). On the contrary, we do not detect Cl^–^ in the film exposed to NaCl.

SIMS analysis allowed us to track the presence of ions throughout the film, up to a depth of 100 nm, from the surface to the substrate. Figure 3e shows Na^+^ signals throughout the films after exposure to each electrolyte, except NaCl, supporting the XPS findings that Na^+^ infiltrates the bulk of the films in all electrolytes except in NaCl, where cations are excluded. Figures 3f and S6 display SIMS curves of the films examined for each anion before and after electrolyte exposure. We observed anion signals throughout the bulk of the films for all electrolytes, except for Cl^–^ from NaCl. Notably, for NaPSS, we detect Na^+^ signals but no PSS^–^, confirming that the polyanion remains on the film surface, while the cation penetrates the bulk. On the other hand, the films immersed in Na_2_SO_3_ present a peculiar case. SIMS measurements suggest that SO_3_^2–^ is excluded from the bulk but appears only in the first few monolayers of the P-100 film (Figure S6), which was the result of the XPS surface analysis.

Overall, the most significant anion infiltration in P-100 films occurs when the films are exposed to NaClO_4_, followed by NaBr, and then Na_2_SO_3_. In contrast, all ions of NaCl and PSS^–^ of NaPSS are excluded from the films’ bulk. A study on monovalent anions argues that their charge density controls their Donnan and dielectric exclusion in a charged polymeric matrix.^28^ Consequently, monovalent anions with the highest charge density, such as Cl^–^, tend to be repelled and remain in the electrolyte, whereas those with lower charge density, such as ClO_4_^–^, can diffuse in the polymer’s bulk. In P-100 films, the polar EG side chains render the films electronegative due to the ether groups, suggesting that co-ions (anions) are excluded from infiltrating the films following the increased surface charge density of Cl^–^ > Br^–^ > ClO_4_^–^. This exclusion follows the DSPM-DE model and is confirmed by our XPS and SIMS data.

For Na_2_SO_3_, dielectric exclusion and the Donnan effect should theoretically exclude the divalent SO_3_^2–^ anion from the hydrated films.^30^ However, we find these ions within the film. One possible explanation for this behavior is the high pH of the Na_2_SO_3_ solution (pH = 9.8). Although we demonstrated above that pH value did not affect the OECT performance, it can influence the strength of electrostatic interactions between anions (SO_3_^2–^) and the membrane (P-100).^28^ The presence of hydroxide ions (OH^–^) in the solution could further affect the ionic distribution and exclusion process. Due to the electronegativity and lone pairs on its oxygen atoms, SO_3_^2–^ can engage in dipole interactions and act as a hydrogen bond acceptor when intereacting with hydrogen-donating molecules. The negative charge is delocalized across the oxygen atoms, allowing dipole–dipole interactions with the polar ether groups of the EG side chains. Additionally, excess OH^–^ ions may facilitate hydrogen bonding between SO_3_^2–^ and the membrane’s polar sites, acting as a bridge that reduces electrostatic repulsion. The high pH conditions may also lower the membrane’s surface charge density, further enabling the anions to approach and interact with the membrane. These combined effects likely allow SO_3_^2–^ ions to penetrate the first few nanometers of the membrane, despite electrostatic considerations.

Note that in a binary 1:1 electrolyte, cations and anions maintain electroneutrality in bulk solution by having equal concentrations. Within the film, however, the local ion distribution is influenced by the negative polarity of the film surface,^26^ fostering ion–dipole interactions resulting in a higher concentration of cations compared to anions. The polar EG side chains preferentially interact with cations through ion–dipole interactions, increasing their affinity for the film. As a result, within the swollen film, cations are expected to be more enriched relative to mobile anions. As cations accumulate near the membrane, electroneutrality is maintained through the redistribution of anions in solution. Following the trend in anion infiltration, ClO_4_^–^ > Br^–^ > Cl^–^, we anticipate the Na^+^ signal to follow the same pattern. Indeed, as shown in Figure 3c, the Na^+^ signal is highest with X = ClO_4_^–^, followed by a weaker Na^+^ signal with X = Br^–^. The absence of a Na^+^ signal with X = Cl^–^ suggests that the concentration of cations in the film is negligible as they remain in the electrolyte. Further validation of our hypothesis comes from the open-circuit voltage (OCP) measurements of P-100 films immersed in various electrolytes (Figure S7). The OCP is positive in the binary electrolytes and negative for the nonbinary electrolyte Na_2_SO_3_. Using these OCP values, we extracted the diffusion potential (*E*_Diffusion_) of P-100, which indicated a preferential Na^+^ infiltration into the films, with *E*_Diffusion_ > 0 for the binary electrolytes (NaBr, NaClO_4_, and NaPSS). In contrast, negative *E*_Diffusion_ values for Na_2_SO_3_ suggest preferential anion infiltration (see Supporting Information, Discussion 3 and Table S1).^36,37^ In the case of NaCl, the negative surface charge density of P-100 governs the polarity of *E*_Diffusion_, leading to a negative value. These findings are consistent with our proposed ion infiltration mechanism and support the idea that interactions between electrolyte ions and side-chain-bearing OMIECs can be modeled analogously to NF membranes using DSPM-DE.

**Figure 4:**
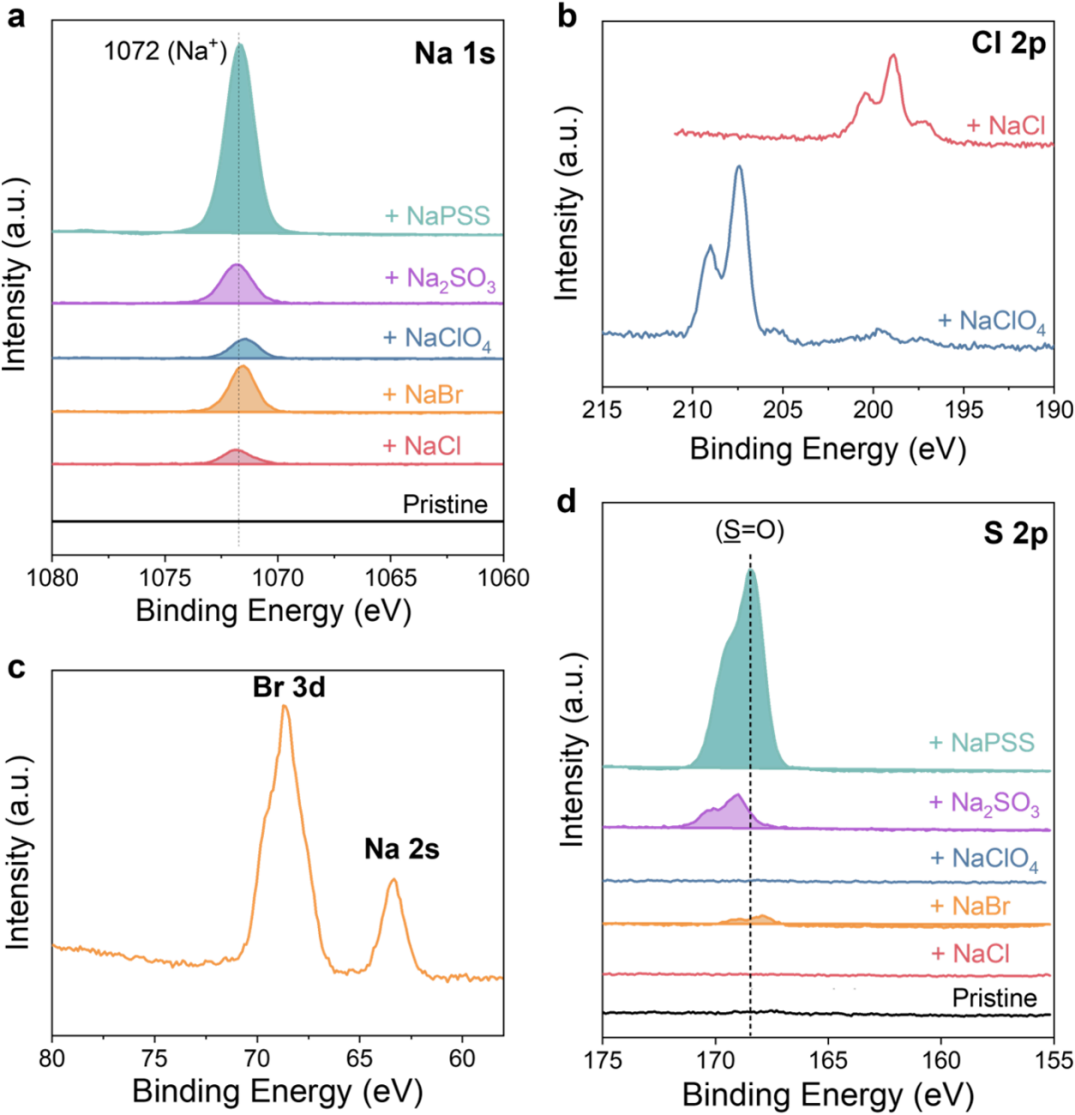
High-resolution XPS spectra of the BBL films before and after immersion in different electrolytes. (a) Na 1s, (b) Cl 2p (NaClO_4_ and NaCl), and (c) Br 3d (NaBr). The presence of the corresponding peaks indicates ion accumulation on the BBL film surface regardless of the electrolyte identity. (d) S 2p signal showing the S=O contribution of PSS^–^ and SO_3_^2–^ anions, suggesting their respective accumulation on the BBL film surface.

In contrast to the P-100 films, XPS data reveals that anions (except PSS) accumulate and infiltrate BBL films regardless of the electrolyte used, including NaCl (Figures 4 and S8–S9). Unlike P-100, BBL films lack polar side chains, which leads to an absence of ion exclusion and thus insensitivity of the performance of the OECT to the electrolyte type. We propose that the presence and nature of the side chains in P-100 films, specifically their polar and electronegative characteristics, render these films comparable to those of NF membranes. This hypothesis is consistent with our steady-state and stability measurements, which showed that BBL OECTs were largely unaffected by the type of electrolyte, while P-100 OECTs exhibited high sensitivity, particularly when comparing electrolytes with monatomic versus polyatomic anions. These findings underscore the critical role that side chains play in determining whether an OMIEC behaves similarly to an NF membrane, which in turn impacts its compatibility with a wide range of electrolytes during operation.

### Hydration due to Passive Ion and Water Uptake

**Figure 5:**
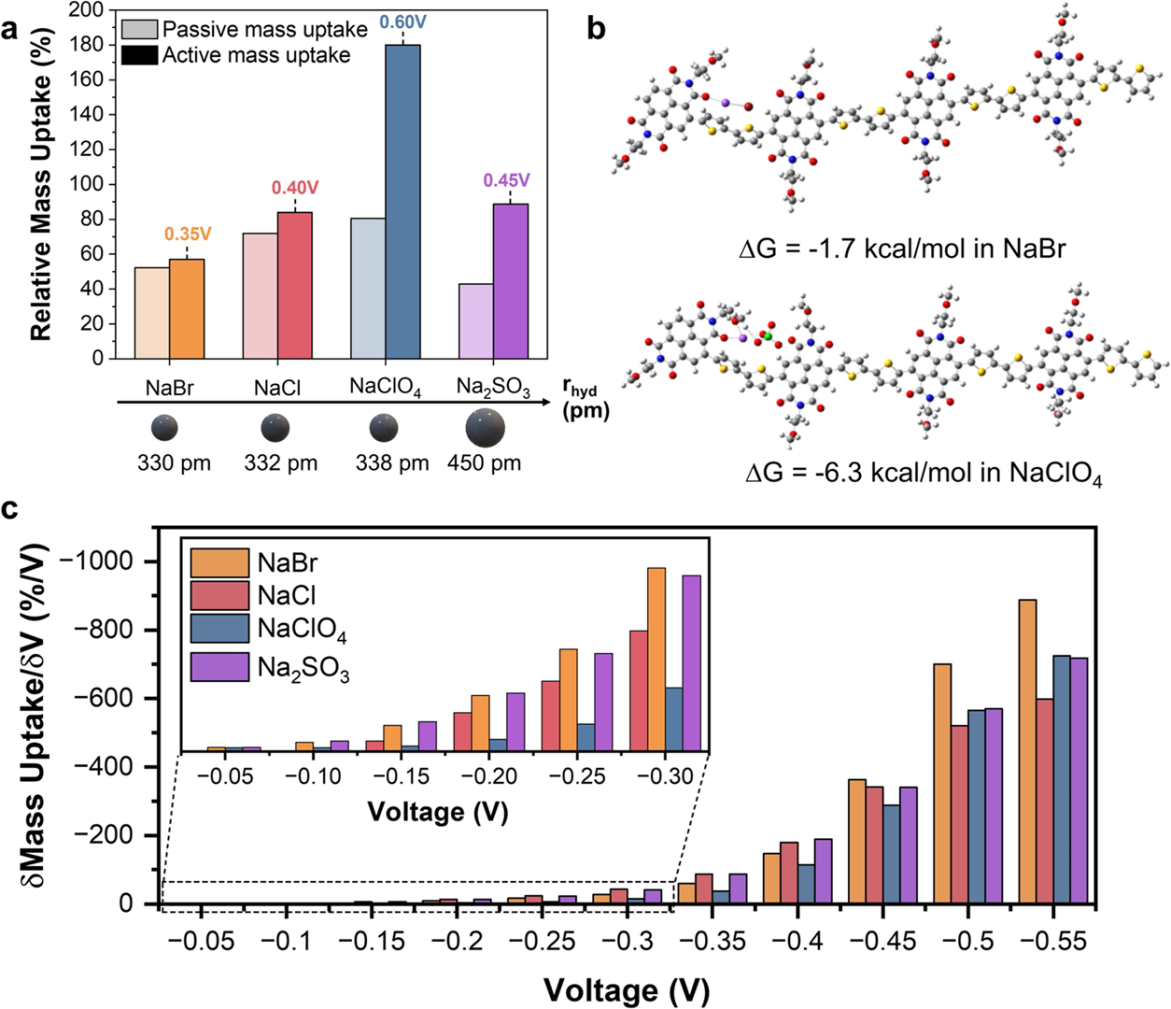
(a) Passive and total active relative mass uptake under electrochemical doping at the potential yielding the *g*_m,max_ of the films in each electrolyte measured with EQCM-D. Relative mass uptake percentages were calculated for the respective P-100 dry films. We also show the corresponding anions’ hydrated radii *r*_hyd_. (b) Electrostatic interactions between NaBr (top) or NaClO_4_ (bottom) and P-100 tetramer. Interaction energies, Δ*G*, are given in kcal/mol. (c) Active mass uptake rate as a function of applied doping potential. All measurements were performed in degassed electrolytes to avoid the influence of oxygen.

We next used the QCM-D to study the passive (diffusion-based) mass uptake of P-100 films exposed to various electrolytes. Knowing the size of the ions (*r*_Hyd_),^38−40^ the solvation shells (see the MD calculations in Figures S10–S13 and Supporting Information, Discussion 4), and the information we reached above regarding which ions diffuse into the film, we can now understand the origin of the passive mass uptake of the P-100 films. In the undoped state, P-100 films uptake different mass percentages when exposed to the electrolytes in the following order of anions: SO_3_^2–^ < Br^–^ < Cl^–^ < ClO_4_^–^ (Figure 5a). The films in NaBr, NaClO_4_, and SO_3_^2–^ show a passive mass uptake directly proportional to the anion infiltration content as determined by XPS and SIMS (SO_3_^2–^ < Br^–^ < ClO_4_^–^). The higher mass uptake of the polymer with NaClO_4_ compared to NaBr cannot be explained by the differences in the size of the hydration shells, as NaClO_4_ has a slightly smaller hydration shell. The mass uptake differences in NaClO_4_ and NaBr seem to increase with the size of the anions (ClO_4_^–^ > Br^–^). However, although the divalent SO_3_^2–^ has the largest size with *r*_Hyd_ = 450 pm, the passive mass uptake of the films in Na_2_SO_3_ is the lowest (ca. 43%). Note that the degree of ion dissociation is identical for the Na^+^ salts with ClO_4_^–^, Br^–^, and Cl^–^ in water, while Na_2_SO_3_ does not undergo complete dissociation in water (Supporting Information, Discussion 4).^41−43^

Importantly, although we did not find NaCl ions in the film through XPS and SIMS measurements, the film uptakes significant mass in NaCl. We suggest that the passive mass uptake observed with X = Cl^–^ is associated with water infiltration rather than ionic charge diffusion via osmotic expansion.^34^ Overall, the passive mass uptake values are not correlated with the anion size or hydrated radius but rather with specific interactions of these anions that contribute to the infiltration ability of the particular anion. We conducted DFT calculations to investigate the interaction energies between the selected electrolytes and P-100 (Figure 5b). The results indicate that the anions interact particularly with the side chains, and the interaction energy between NaClO_4_ and P-100 is more favorable compared with NaBr with a 4.6 kcal/mol difference (significantly beyond the statistical error margin), in agreement with the observed differences in mass uptake in these electrolytes. We also found a relatively low diffusion coefficient for ClO_4_^–^, which may entrap it inside the polymer film (Table S2).

### Relative Mass Uptake during Biasing and Its Impact on Device Performance

Next, we designed a QCM-D experiment to understand how variations in passive ion uptake can affect electrochemical doping. We investigated the extent to which P-100 films uptake mass during electrochemical doping in each electrolyte, calculating the total active mass change at the voltage yielding their respective *g*_m,max_ values (Figure 5a, darker bars). The film exhibits varying degrees of active mass uptake, depending on the electrolyte. Films exposed to NaBr demonstrate the least active mass change at 57%, while those in NaClO_4_ exhibit significantly higher mass uptake at 180%. The mass uptakes in NaCl and Na_2_SO_3_ reach 84 and 89%, respectively. Notably, the films that experience more relative mass uptake display consistently lower *g*_m,max_ values and higher *V*_Th_.

To better understand the trend in active mass uptake and its relationship to electrolyte-dependent OECT performance, we plotted the relative change in mass with respect to a change in voltage as a function of the increasing doping potentials applied, which we refer to as the “mass uptake rate”, i.e., a measure of the film’s sensitivity to doping voltage change (Figure 5c). At potentials below −0.2 V vs Ag/AgCl, the mass uptake is insignificant in NaClO_4_ and NaBr. The mass uptake rates in NaCl at this low voltage range are similar to those in Na_2_SO_3_ but much higher than those in NaBr and NaClO_4_. At potentials above −0.45 V vs Ag/AgCl, the mass uptake rate in NaBr becomes much larger than all other electrolytes. Notably, the mass uptake rate in NaClO_4_ becomes significant only at much higher potentials. The mass uptake rate in NaClO_4_ demonstrates a more gradual change as voltage increases, eventually surpassing the mass uptake rate in NaCl at potentials greater than −0.5 V vs Ag/AgCl. Similarly, the mass uptake rate in Na_2_SO_3_ exceeds that of NaCl at potentials above −0.5 V vs Ag/AgCl.

The differences in total mass uptake and mass uptake rates upon electrochemical doping can be attributed to the varying cation and anion transport mechanisms within the polymer film. We identify two concomitant ion transport processes in P-100 films during electrochemical doping: (1) cation injection and (2) anion expulsion (if applicable). When a positive gate bias is applied, the hydrated cations are transported toward the film while anions are expelled through electromigration. We suggest that the observed mass uptake behavior and the resulting OECT performance are driven by competition between these two processes. For chaotropic anions like Br^–^ and ClO_4_^–^, the initial doping-induced mass uptake rates are low, as the films must first expel the anions. Only after these anions are ejected do the mass uptake rates reach their maximum with cation uptake. In contrast, NaCl has an increase in the mass uptake rate starting at much lower voltages because charge compensation is governed exclusively by cation uptake, with no pre-existing anions within the film before doping. The similarity in mass uptake rates at lower voltages between NaCl and Na_2_SO_3_ suggests that the hydration process in Na_2_SO_3_ is also dominated by cation uptake rather than SO_3_^–^ expulsion (recall that XPS data revealed SO_3_^–^ only at the uppermost surface of the polymer films). At higher potentials, after a few anions are expelled, the mass uptake rates of the film in Na_2_SO_3_ surpass that of NaCl. These findings also support the differences in *V*_Th_ values of the OECTs operating in NaBr, NaCl, and NaClO_4_. Interfacing all electrolytes (except NaCl), the film has Na^+^ inside, yet the onset voltage is associated with how easy it is to eject the anions based on their size (and diffusion coefficient) or infiltration depth. The lowest *V*_Th_ for the OECTs in NaBr (0.18 V) is due to the readily available Na^+^ cations facilitating doping via rapid Br^–^ expulsion. In NaCl, the slightly higher *V*_Th_ (0.21 V) is likely due to the cost of disturbing the electrolyte’s electroneutrality. In NaClO_4_, the highest *V*_Th_ (0.35 V) reflects the significant energetic cost of expelling the polyatomic anions, which strongly interact with the film. Finally, in Na_2_SO_3_, where cation injection dominates anion expulsion, the moderately high *V*_Th_ (0.28 V) aligns with the balance of these processes in the channel.

In parallel, we sought to correlate the *V*_Th_ with the cyclic voltammograms (CVs) of the P-100 film in the different electrolytes. Figure S14 displays the P-100 CVs, exhibiting features—such as reduction onsets, current peaks, half-wave potentials *E*_1/2_^I/II^, and redox couples with slight variations depending on the coanion (Table S3). All CVs, except those in NaPSS, show two distinct redox waves around −0.4 V (red1) and −0.6 V (red2), which we attribute to the stepwise formation of the NDI radical anion (see Supporting Information, Discussion 5 and Figures S15–S16).^44^ In electrolytes containing polyatomic anions (ClO_4_^–^, PSS^–^, and SO_3_^2–^), the red1 peak is weak and shifted to more negative potentials, while the red2 peak is more prominent, indicating a delayed doping onset, in support of the *V*_Th_ trend in the OECTs. On the other hand, monatomic anions (Br^–^ and Cl^–^) result in earlier doping onset, with prominent red1 and red2 reduction waves, suggesting reduction of the chains at low voltages. Comparing NaCl and NaClO_4_, Cl^–^ ions are excluded from the film, while ClO_4_^–^ ions infiltrate. Consequently, in NaCl, efficient charge compensation occurs through direct Na^+^ injection at lower potentials (leading to pronounced red1). In contrast, polymer reduction in NaClO_4_ begins only after the convection-driven transport of Na^+^ ions and the expulsion of ClO_4_^–^ anions, leading to a subdued red1 signal. A negative shift with NaClO_4_ [*E*_1/2_^I^ = 60 mV] compared to that with NaCl indicates that polymer reduction requires higher potentials (Table S3). A similar rationale can be applied to the other electrolytes. For NaBr, ejecting the less abundant monatomic anion Br^–^ is energetically more favorable than that of the polyatomic anion ClO_4_^–^, leading to the trend [*E*_1/2_^I^ in NaCl] < [*E*_1/2_^I^ in NaBr] < [*E*_1/2_^I^ in NaClO_4_]. In Na_2_SO_3_, the adsorption of SO_3_^2–^ on the P-100 surface, combined with the weak degree of dissociation of this salt as predicted by MD simulations, hinders early reduction, shifting *E*_1/2_^I^ to more negative values. For NaPSS, the formation of a polymeric anion layer on top of the P-100 film impedes cation injection, thereby suppressing the first redox wave.

### The Processes during the Anion-Dependent Electrochemical Doping of the n-Type OMIEC

**Figure 6:**
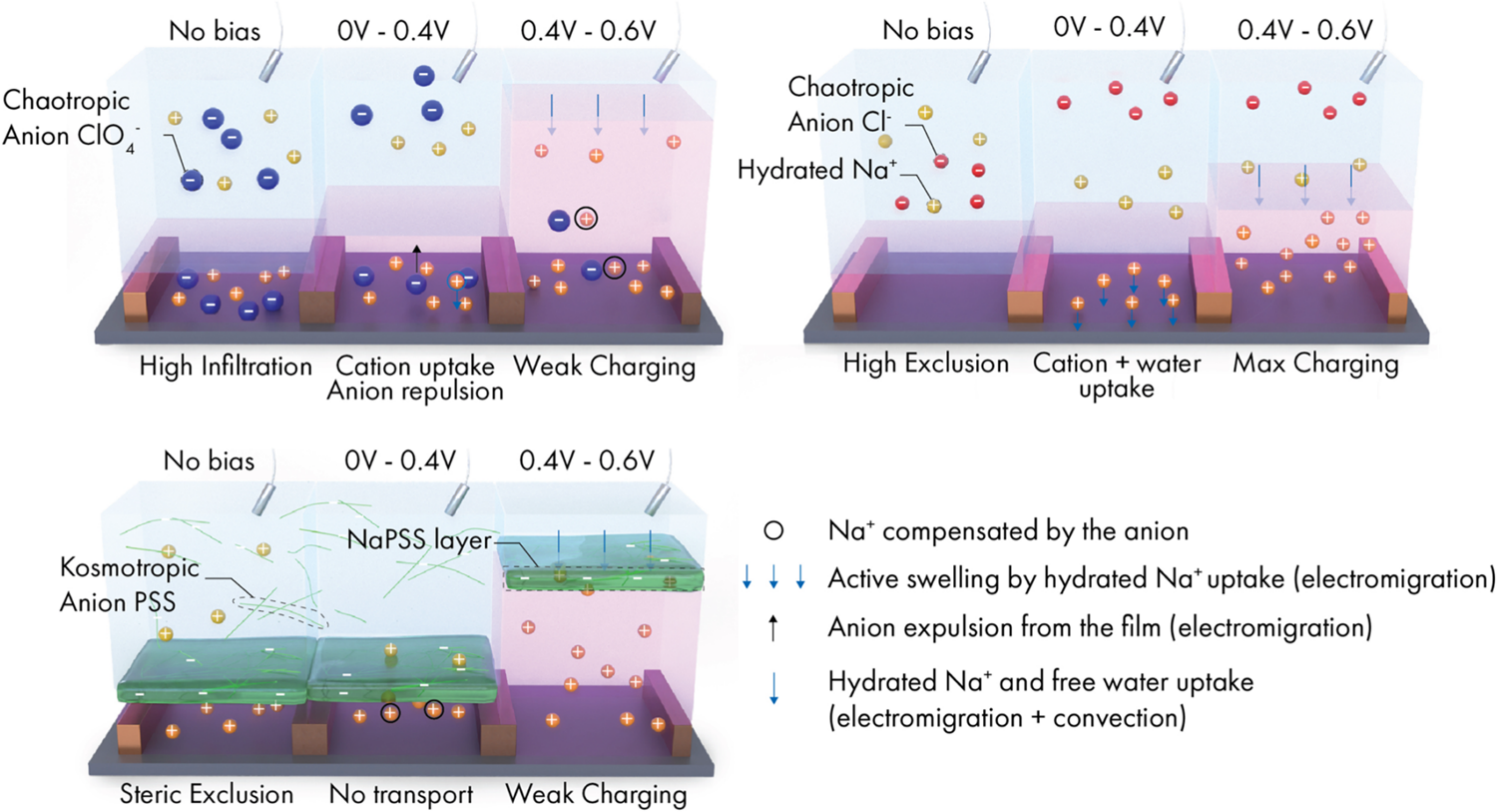
Schematics illustrating the three stages of electrochemical doping in the OECTs operated in NaCl, NaClO_4_, and NaPSS. Each panel corresponds to the channel doped in a specific electrolyte and is divided into three stages where there is no bias, at *V*_G_ = 0–0.4 V and *V*_G_ = 0.4–0.6 V.

Our findings underscore the importance of anion-polymer interactions and ionic pair dissociation in determining the ion diffusion, anion expulsion, and, consequently, the electrochemical behavior of the OMIECs. In Figure 6, we provide a schematic summary of the key processes occurring in the film within three very different electrolytes, divided into three main stages:

1. In the absence of bias: When the film is exposed to the electrolyte, it passively swells with water molecules and, if there are favorable interactions, with hydrated ions. In this stage, ion transport occurs through diffusion, driven by the ionic concentration gradient across the polymer film interface. However, ions may interact with the electronegative polar side chains and the film’s adsorbed charges, creating exclusion effects based on the ion’s charge (Donnan effect), hydration shell, the solvation barrier between the two media (dielectric effect), and steric effects. During this stage, ions either diffused into or are excluded from the film.
2. At low voltages (<0.4 V): If anions are excluded from the film, doping occurs via cation injection only. If ions are not excluded and are already present in the film, electrochemical doping involves the expulsion of anions alongside cation injection. The ejection of anions in these electrolytes results in low mass uptake rates. If the membrane is fully hydrated but has no ions or if it has many ions taken up via diffusion, an osmotic pressure gradient can drive water movement into or through the membrane. Ions dissolved in the water may be carried along with this flow, creating a convection-like transport mechanism.
3. At high voltages (0.4–0.6 V): In this stage, most anions are expelled from the film, and cation injection becomes the dominant charge compensation process. Cations can infiltrate deeper, possibly reaching crystalline or more dense domains, maximizing electronic charge compensation.

In NaCl and NaPSS, ions are predominantly excluded, with Cl^–^ ions excluded due to strong Donnan and dielectric exclusion effects, and PSS^–^ ions excluded by steric hindrance. In the initial doping window (<0.4 V), Na^+^ injection to the film is dominant in NaCl, while the polymeric PSS^–^ hinders cation movement. At higher bias (>0.4 V), active mass uptake increases with more Na^+^ uptake. The PSS^–^ layer is expected to overhydrate at these high doping potentials, allowing cations to be injected. However, during the OECT cycling, PSS^–^ anions can become trapped on the P-100 surface, compromising device stability. In contrast, OECTs remain stable in NaCl.

Anions in NaBr and NaClO_4_ can diffuse into the film. The film’s hydration level is controlled by the number of infiltrated ions. ClO_4_^–^ is more present in the films than Br^–^ due to fewer exclusion effects and more favorable anion-polymer interactions, resulting in greater passive mass uptake in NaClO_4_. Given the strong polymer-ion interactions in NaClO_4_, the first doping window, dominated by both electromigration and convection events, requires a higher energetic cost for expelling the polyatomic anions, while the expulsion of anions is more efficient for the less abundant monatomic Br^–^. OECTs operating in NaBr have thus a lower *V*_Th_ and reach higher *g*_m_ values at a low bias. At higher potentials, doping in both electrolytes is governed by cation injection. The contrast in mass uptake is crucial for determining the characteristics of the OECT at higher potentials, resulting in superior device performance for channels operating in NaBr compared to those operating in NaClO_4_. Polyatomic anions negatively impact device stability, likely due to anionic trapping during device cycling.

In Na_2_SO_3_, ion infiltration is minimal and mostly confined to the surface, making the behavior somewhere between NaCl and NaPSS. Na_2_SO_3_ salt does not fully dissociate and has a high pH at the concentration used, altering the conditions of the DSPM-DE model. Additionally, hydrogen bonding is more likely to occur with this electrolyte. Consequently, SO_3_^2–^ anions are preferentially located on the film surface compared to Na^+^ cations, leading to a shift in *V*_Th_ to higher potentials. While the films exhibit low mass uptake levels in both the passive and active states, the facile cation injection improves device performance compared to that of NaClO_4_. Nevertheless, the polyatomic and strongly kosmotropic nature of this divalent anion persists in causing device instability.

## Conclusions

This study sheds light on the often-overlooked role of noncompensating ions in electrochemical doping, offering a comprehensive understanding of how electrolyte components, water, cations, and anions can affect the performance of n-type OECTs. By investigating five Na^+^-based electrolytes and applying the DSPM-DE model, we demonstrate the significant influence that anions have on n-type device steady-state performance and operational stability if the mixed conductor contains polar side chains. We find that the polymer lacking hydrophilic or glycolated side chains, i.e., BBL, is minimally impacted by anions, underscoring the critical role of polar side chains in governing anion interactions. The various experimental methods employed ranging from DFT calculations and hydration measurements to elemental analysis via XPS and SIMS present a cohesive and complementary view of the system. Our findings indicate that chaotropic anions with high charge density are excluded from film infiltration, whereas lower-charge-density anions are more readily incorporated into undoped films. Voltage-dependent mass uptake rates further support the role of anion expulsion and cation injection in the doping process. Cyclic voltammetry curves reveal that in cases where anions are passively incorporated, electrochemical doping exhibits a delayed onset due to the necessity of anion expulsion, resulting in a higher *V*_Th_ in OECTs. Conversely, when anions are excluded, the doping efficiency is dictated primarily by direct cation injection, enabling doping at lower potentials. Our analysis demonstrates that the passive infiltration of ions in the absence of bias fundamentally shapes the polymer’s subsequent electrochemical behavior by establishing the ionic distribution and interactions within the film. This distribution governs the sequence of ion expulsion and uptake during doping, supporting the conclusion that anion expulsion precedes the effective cation incorporation when passive infiltration occurs. Across all electrolytes studied, polyatomic anions consistently compromise device stability.

Our results suggest that tailored anion exclusion strategies could enhance device stability and optimize the performance of the OECT performance. This understanding may be more crucial for vertically configured OECT devices (i.e., channel vertically placed between the source and the drain) where the electrolyte/OMIEC wetting dictates performance.^45^ This is the first study to extend the concepts of NF and DSPM-DE to semiconducting polymers, paving the way for future research to further elucidate the interaction between OMIECs and electrolytes. By advancing this knowledge, we aim to inspire the development of the development of the OMIECs together with the electrolyte as a system, suggesting a new design space for optimizing electrochemical device performance and enhancing their functionality.

## Supporting Information Available

Materials and methods; OECT characterization; XPS and SIMS depth profiling signals of polymer films before and after immersion in various electrolytes; MD calculations of ion solvation shells; electrochemical characterization (EIS, CV, in situ conductivity, electrochemical UV–vis); discussions on the Hofmeister series, performance of OECT devices gated with a polarizable electrode (Pt), qualitative interpretation of open-circuit potentials, the role of degree of dissociation and analysis of hydrated radius of ions, and stepwise formation of the radical anion (PDF)
