# Supplementary material for: Impact of Noncompensating Ions on the Electrochemical Performance of n-Type Polymeric Mixed Conductors
Source: J Am Chem Soc. 2025 Apr 7;147(15):12523–33. doi: 10.1021/jacs.4c17579 (PMC12006988; doi:10.1021/jacs.4c17579)
Supplement: Supplementary file 1 — ja4c17579_si_001.pdf [file ja4c17579_si_001.pdf]

Supporting Information

Impact of non-compensating ions on the electrochemical performance of n-type polymeric mixed conductors

David Ohayon[1,2,3Y], Amer Hamidi-Sakr[1Y], Jokubas Surgailis[1], Shofarul Wustoni[1], Busra Dereli[4], Nimer Wehbe[5], Stefan Nastase[4], Xingxing Chen[6], Iain McCulloch[6,7], Luigi Cavallo[4], Sahika Inal[1]*

[1] Organic Bioelectronics Laboratory, Biological and Environmental Sciences and Engineering Division, King Abdullah University of Science and Technology (KAUST), Thuwal 23955-6900, Saudi Arabia.

[2] Institute for Functional Intelligent Materials, National University of Singapore, Singapore, 117544 Singapore

[3] Departments of Chemistry and Chemical & Biomolecular Engineering, National University of Singapore, Singapore, 119077 Singapore

[4] Catalysis Research Center, Physical Sciences and Engineering Division, KAUST, Thuwal 23955-6900, Saudi Arabia.

[5] Imaging and Characterization Core Lab, King Abdullah University of Science and Technology (KAUST), Thuwal, 23955, Saudi Arabia

[6] KAUST Solar Center, Physical Sciences and Engineering Division, KAUST, Thuwal 23955-6900, Saudi Arabia.

[7] Department of Chemistry, University of Oxford, Oxford OX1 3TF, U.K.

[Y] These authors contributed equally to this work.

Corresponding author: sahika.inal@kaust.edu.sa

Materials and Methods

Sample preparation

All polymer films (on ITO and OECTs) were spin-coated from a chloroform solution at a 5 mg/mL concentration. The spinning speed was 1500 rpm, the acceleration was 300 rpm/s, and the overall spinning time was 120 seconds. Devices were stored in a vacuum for 2 hours after casting.

OECT fabrication

The devices were fabricated according to the parylene-C lift-off method reported previously.[1] Briefly, glass wafers were cleaned via sonication in an acetone and isopropyl alcohol solution and dried with N_2. Connection pads and interconnects were deposited through a lift-off process using photolithographic patterning of positive photoresist (S1813). A subsequent metal deposition via sputtering of Cr (10 nm) and Au (120 nm) and metal lift-off using acetone defines the Au lines. The first layer of parylene C (2 µm), deposited together with a small amount of 3-(trimethoxysilyl)propyl methacrylate (A-174 Silane) to enhance adhesion, acts as an insulator to prevent disturbing capacitive effects at the metal liquid interface. Subsequently, an antiadhesive layer was spin-coated using a dilution of industrial cleaner (2 wt %, Micro-90), and a second parylene-C sacrificial layer (2 µm) was deposited. To define the contact pads and the channel of the OECT, a second photolithographic patterning step using a thick positive photoresist (5 µm, AZ9260) and AZ developer was used to protect the parylene-C layers from a subsequent plasma reactive ion etching step. After spin coating, and peeling the parylene-C sacrificial layer, the polymer layers defined the channel dimensions. The thickness of the channels was measured using a DEKTAK 150 stylus profilometer. The devices have planar dimensions of L=10 µm and W= 100 µm.

OECT characterization

OECTs were characterized using a dual-channel source-meter unit (NI-PXI) with a custom-written control code in LabVIEW. All measurements were performed using an Ag/AgCl pellet (D =2mm × H = 2 mm; Warner Instruments) or a Pt coil as the gate electrode. The electrolyte solutions were contained in a PDMS well over the OECTs, and the electrolyte volume was maintained constant (200 µL) for all measurements. The OECT figures of merit were calculated according to recommended procedures.[1] V_{Th} was obtained from the maximum slope of the $\sqrt{I_D}$ vs. V_G curve. The material figure of merit μC^* was estimated using equation (1) (see manuscript) in the saturation regime and maximum g_m. The volumetric capacitance C^* was calculated from the capacitance equation: $C = \frac{1}{2\pi f \times |Img(Z)|}$ at f=1 Hz, obtained for films doped at an offset voltage corresponding to the respective V_G for maximum g_m. The electronic charge mobility μ was calculated by dividing μC^* over the calculated volumetric capacitance. The On:Off ratio was obtained by calculating $I_D(V_G = 0.6\text{V})/I_D(V_G = 0\text{V})$ at $V_D = 0.6$V.

Electrochemical characterization

Electrochemical measurements were performed using a potentiostat (Metrohm Autolab) with a three-electrode configuration. Cyclic voltammograms at ambient temperature were recorded

in a three-electrode setup using an Ag/AgCl reference electrode and a Pt foil counter electrode. Electrochemical impedance spectroscopy (EIS) measurements were conducted to determine the polymer's electrochemical capacitance in 0.1M Na^+ electrolyte solutions. The polymer films were spin-coated on the ITO working electrodes with a surface area of 2 cm^2. Measurements were performed at a DC offset potential and a sinusoidal AC amplitude of 10 mV, spanning a frequency range from 10 kHz to 0.1 Hz. The fits were generated using EC-Lab software V10.44. The results were fit using $R_s(R_p||C)$ equivalent circuit where R_s is the electrolyte resistance, and R_p and C describe the polymer film's resistance and capacitance, respectively. The effective capacitance C_{eff} was calculated using $C_{eff} = \frac{1}{2\pi f \times |Img(Z)|}$. The capacitance values generated by the $R_s(R_p||C)$ fit match those calculated from C_{eff} (at f=1 Hz). The final capacitance values (obtained from the EIS fits) were normalized for the film volume to determine the volumetric capacitance (C*).

Chronopotentiometric measurements showing open-circuit potentials (OCP) of P-100 thin films cast on ITO substrates of area 2 cm^2 are recorded for films exposed to the five electrolytes. The open circuit potential values are reported after the system reaches stability ($\frac{\partial V}{\partial t}< 10^{-6}$ V/s) in the passive state before any electrochemical doping is exerted on the system and after CV cycling.

Electrochemical UV-Vis spectroscopy (E-UV-Vis)

E-UV-Vis measurements were performed using UV-1601 UV-VIS Shimadzu UV-Vis spectrometer coupled with a Metrohm autolab PGSTAT101 potentiostat in a three-electrode setup. The polymer was deposited on ITO-coated glass slides, which acted as the working electrode. The films were placed in a MM Spectro-EFC, SMA 905, 1.75 mL with optical windows purchased from Redox.me. A Pt mesh was used as the counter electrode, an Ag/AgCl electrode as the reference electrode, immersed in 0.1M Na^+ electrolyte solutions. A background measurement was taken with a clean bare ITO substrate. The indicated voltages were applied for 10 seconds until the current stabilized before measuring the spectrum.

X-Ray Photoelectron Spectroscopy characterization

To characterize the elemental composition of the films, X-ray Photoelectron spectroscopy (XPS) was carried out using a Kratos Axis Supra instrument equipped with a monochromatic Al Kα X-ray source (hν = 1486.6 eV) which was operated at a power of 150 W and under UHV (in the range of $\sim 10^{-9}$ mbar). All spectra were recorded in hybrid mode using electrostatic and magnetic lenses. The survey and high-resolution spectra were acquired at fixed S-5 analyzer pass energies of 80 and 20 eV, respectively. The obtained spectra were calibrated using the reference C 1s at 284.8 eV. The spectra were fitted using XPSPeak4 software with Gaussian and Lorentzian methods, while the background was subtracted using the Tougaard method. The P-100 and BBL films were exposed to the various electrolytes for 1 hour and then dried with a Nitrogen flow. All films were compared with a Pristine film hydrated with Milli-Q purified water.

Secondary Ion Mass Spectrometry (SIMS)

Depth profiling experiments were performed on a Dynamic SIMS instrument from Hiden Analytical Company (Warrington, UK) operated under ultrahigh vacuum conditions, typically

10^{-9} Torr. A continuous Ar^+ beam of 4 keV energy was employed to sputter the surface while the selected ions were sequentially collected using a MAXIM spectrometer equipped with a quadrupole analyzer. The raster of the sputtered area is estimated to be 750 × 750 μm^2. To avoid the edge effect during depth profiling experiments, it is necessary to acquire data from a small area located in the middle of the eroded region. Using adequate electronic gating, the acquisition area from which the depth profiling data are obtained was ~75 × 75 μm^2. The P-100 films were exposed to 0.1 M of Na^+ electrolytes for 1 hour and then dried with a Nitrogen gun.

Electrochemical quartz crystal microbalance with dissipation monitoring (EQCM-D)

E-QCMD is a microbalance that monitors in-situ fluctuations in the mass of an electroactive film coated on a piezoelectric quartz crystal while subjected to electrochemical reactions. The principle in which the EQCM-D functions is by measuring the changes in the oscillation frequency (*f*) as well as energy dissipation (*D*) of the quartz crystal; a change in the frequency indicates a change in the total mass attached to the substrate, while a change in dissipation indicates a change in the viscoelasticity of the attached sample. Thus, in thin films, a decrease in (*f*) signifies an uptake of mass caused by an increase in the film's hydration, while an increase in (*D*) indicates that the film has become softer. However, EQCM-D cannot distinguish between an increase in mass caused by the film's hydration or caused by the adsorption of other polymeric species (e.g., macromolecules) to the substrate. This discrimination between the origin of the energy dissipation is vital when studying the effect of polymeric aqueous electrolytes, such as NaPSS, on the films' hydration. As shown in the manuscript section, these polymeric chains are adsorbed on top of the film; consequently, the measurements discussed with EQCM-D to characterize hydration did not include the NaPSS.

Measurements were performed using the QSense analyzer, a QSense Electrochemistry Module (QEM 401), and gold with titanium adhesion layer sensors (QSensors QSX 338) from Biolin Scientific in deoxygenated electrolytes bubbled continuously with Nitrogen gas. The analyzer measured the shift and dissipation of the 1st, 3rd, 5th, 7th, $9^{th,}$ and 11th frequency harmonics, which provide information about the changes in sample mass and softness. First, the bare sensors were measured in air and after introducing the electrolytes. The electrolyte resulted in a significant shift in the QCM-D signals due to the change in media density inside the chamber, which needs to be considered when calculating polymer swelling associated with relative mass uptake. Note that since the density of the polymer film is not constant throughout the study, and this can complicate the interpretation of swelling values, we thereby only refer to the polymer's relative mass uptake for our analysis. After acquiring QCM-D baseline signals in dry and wet conditions, the measured sensor was coated with the polymer film and placed back into the analyzer. The QCM-D signals were re-recorded in the dry state and with electrolyte after the *f* and *D* signals were stabilized (i.e., $\Delta f < 0.1$ Hz / 5 min). The QSoft software function "stitch data" provided the difference between the *f* and *D* signals before and after the sensor was coated with a polymer film in dry states and electrolytes. This difference was used to calculate the areal film mass in dry and swollen states, using the Sauerbrey equation:

$$\frac{\Delta m}{A} = -\Delta f_n \frac{\rho_q V_q}{2 f_0^2 n} \approx \frac{-\Delta f_n}{n} \approx 17.9 \text{ ng/cm}^2 \qquad \text{(Equation S1)}$$

Where Δf_n is the frequency shift of the nth overtone, A is the 'sensor's active area, ρ_q is the density of quartz, V_q is the shear wave velocity in quartz, f_0 is the fundamental frequency, and n is the overtone number. Film thickness was calculated by dividing the calculated areal mass by film density.

Electrochemical doping was performed using an Autolab PGstat128N potentiostat coupled with a QSense electrochemistry module (QEM 401). The integrated three-electrode setup comprised an Ag/AgCl reference electrode, a Pt counter electrode, and the polymer-coated Au QSensor acting as the working electrode with an active electrochemical area of 0.7854 cm^2. Physical modeling of the measured f and D signals was done based on observed film characteristics. Films exhibiting low relative mass uptake and little to no energy losses were considered "rigid" and modeled using the Sauerbrey equation. Whereas films exhibiting higher degrees of relative mass uptake were modeled as Kelvin-Voigt elements, exhibiting viscoelastic properties. Such an element has a complex shear modulus, described as:

$$G^* = \mu + 2\pi i f \eta \quad \text{(Equation S2)}$$

Where $G*$ is the complex shear modulus, μ is elasticity (kg m^{-1} s^{-2}), η is viscosity (kg m^{-1} s^{-1}), and f is the frequency. To calculate the mass changes of the soft film, the complex shear modulus was analyzed and fitted using several frequency overtones using Q-Tools to model the films' relative mass uptake data. The relative mass uptake in **Figure 5** was calculated as the percentage change in mass uptake relative to the dry mass:

$$\text{Relative Mass Uptake} = \frac{M_{total} - M_{dry}}{M_{dry}} * 100\% \quad \text{(Equation S3)}$$

Molecular Dynamics (MD)

Ab initio MD simulations were performed with the CP2K simulation package (version 8.1). The nuclei's dynamics were governed by the Newtonian equations of motion, in which the potential from the Born−Oppenheimer electronic ground state is inserted. The self-consistent field (SCF) energy was evaluated with density functional theory using the revPBE functional with Grimme D3 dispersion corrections and the Gaussian plane wave method that uses a combination of Gaussian basis functions (DZVP−GTH) and plane waves (320 Ry cutoff). The SCF convergence criterion was set to 1×10^{-5} Hartree between SCF iterations. The integration time step was set to 0.5 fs. For the various NaX salts (X = Cl^-, Br^-, ClO_4^-, $SO3_2^-$), a 70 ps MD simulation is carried out in the NVT ensemble at 300 K, with 20 ps equilibration and the following 50 ps representing the main production sample, controlled by a chain of five Nose−Hoover thermostats. The cell volume was fixed to the cell parameters obtained from the calculated density of aqueous solutions of the NaX salts above 0.1 M using experimental constants.[2] Trajectory snapshots are taken every 1 fs of the 70 ps NVT production run. Diffusion coefficients are calculated using the mean square displacement (MSD) versus time relation using the $D = \frac{1}{6}\frac{d<r(t)^2>}{dt}$ formula on the 50 ps trajectory.

Density functional theory (DFT) calculations were applied to calculate the interaction energy between the NaX ions and polymer. As model structures, tetramers, which are capable of accounting for local electronic features of the polymer, were used. DFT calculations were employed in Gaussian16. Full geometry optimizations were performed at the hybrid PBE0 functional using Becke Johnson dispersion correction D3 with a damping function. Geometry optimizations utilized 6-31+G(d) basis set while single point energy calculations were carried out at M06 functional with 6-311+G(d,p) basis set. Solvent corrections in water ($\varepsilon = 80$) were added to the gas-phase geometries using SMD continuum solvation model. The stationary nature of all structures were confirmed by analytic computation of their vibrational frequencies at 298.15 K. All frequencies below 50 cm^{-1} were replaced by 50 cm^{-1} when computing vibrational partition functions.

Supplementary Discussions

Discussion 1. Hofmeister Series

One critical property of an ion in aqueous electrolytes is its hydration level. It is well established that an ion's hydration behavior depends on its surface charge density, and is often represented by the Hofmeister series, categorizing ions into two groups: chaotropes and kosmotropes.[3,4] Typically, Hofmeister anions follow the series $CO_3^{2-} > SO_3^{2-} > SO_4^{2-} > F^- > Cl^- > Br^- > I^- > ClO_4^-$ in terms of their hydration strength.[5-7] The four anions on the left side of the series, known as kosmotropes, are prone to hydration due to more localized valence charges binding to the polar water molecules. The four anions on the right side of the series, known as chaotropes, have less localized valence charges, interacting weakly with water molecules.[5]

As formulated by Collins, the law of matching water affinities describes how ion-ion and ion-surface interactions form based on their respective degree of hydration.[4] Kosmotropic ions have higher affinities to hydrophilic species and surfaces, while chaotropes have weak affinities to hydrophilic species and surfaces. In the electrolytes studied in this work, we associate the PSS^- anion as a kosmotropic ion, keeping in mind that the sulfonate group in PSS^- is very similar to the kosmotropic sulfite anion. Moreover, PSS^- has been shown to display a salting-out effect on methylcellulose, a characteristic behavior of kosmotropes.[8]

Discussion 2. Performance of devices gated with a polarizable electrode (Pt)

In non-polarizable electrodes, such as Ag/AgCl, a steady-state current flows from the gate electrode to the solution without forming an electric double layer. For the polarizable Pt, however, the anions cause a drop in gate potential due to the electric double layer's formation at the Pt/electrolyte interface. Therefore, overall, the devices gated with Pt have lower performance than those with Ag/AgCl in all electrolytes except Na_2SO_3.

In $Na_2SO_{3(aq)}$, the sulfite ion is easily oxidized on the Pt electrode by reacting with molecular oxygen present in the electrolyte to form a sulfite radical or a sulfate ion through one or two 1-electron transfer steps.[9,10] Consequently, this oxidation at the gate electrode causes a faradaic response in this system, lowering the V_{Th} compared to using an Ag/AgCl gate electrode. To confirm that the Faradaic reaction is responsible for the device behavior observed, we designed a new experiment where we evaluate device performance in sodium sulfite (Na_2SO_3) and sodium sulfate (Na_2SO_4). The sulfate ion is stable and does not undergo faradaic reactions at the Pt electrode. The Pt-gated OECT performances in Na_2SO_4 are lower than in Na_2SO_3 (**Figure S4**).

Finally, OECTs in $NaPSS_{(aq)}$ do not turn on when gated with Pt. The polymeric anion's bulky nature causes a large drop in potential at the gate/$NaPSS_{(aq)}$ electrolyte interface, further screening the electric field, and thus diminishing the effective gate potential. Note that for this polarizable gate, the monoatomic anions lead to higher g_m values than polyatomic ones, a similar trend to what we observed with Ag/AgCl.

Discussion 3. Qualitative Interpretation of Open-Circuit Potentials

The Open-circuit potential (OCP) switches polarity in our system depending on the electrolyte. For example, for the sulfite anion, the OPC < 0, in contrast to other electrolytes. According to previous works, the electrochemical potential of a membrane is related to the ion diffusion potential, following this relationship: $E_{Diffusion}$ = OCP – E_{Redox}, where E_{Redox} relates to the unequal potential drop at the electrode-solution interface.[11,12] The polarity of the $E_{Diffusion}$ across an ion-permeable membrane is directly tied to the concentration gradient and the charge of the ions. The OCP reflects the cumulative effect of these ionic movements and their respective diffusion potentials. We calculated $E_{Diffusion}$ by measuring E_{Redox} of the film on ITO electrodes and then subtracting it from the measured OCP values in **Figure S8**, and compiled the values in **Table S1**.

Table S1. The measured OCP and E_{Redox} and the calculated $E_{Diffusion}$

	NaCl	NaBr	$NaClO_4$	NaPSS	Na_2SO_3
OCP (V) | before cycling	0.075	0.152	0.290	0.259	-0.06
E_{Redox} (V)	0.148	0.126	0.136	0.097	0.07
$E_{Diffusion}$ (V)	-0.073	0.026	0.154	0.162	-0.13

The results compiled in **Table S1** show the same polarities for OCP and $E_{Diffusion}$ (except for NaCl), confirming that the preferential presence of ions in the film's bulk dictates the measured OCP polarity, which is positive. NaCl, showing no infiltration of both ions, represents a peculiar case where the P-100 negative charge surface density (ζ-potential) dictates the polarity of the $E_{Diffusion}$. For the other electrolytes, the polarity of $E_{Diffusion}$ indicates a preferential presence of either the anion (if $E_{Diffusion} < 0$ for Na_2SO_3) or cations (if $E_{Diffusion} > 0$ for NaBr, $NaClO_4$, NaPSS). Recall that XPS and SIMS measurements showed higher levels of sodium cations than counter-anions in the surface (XPS) and the bulk (SIMS) of P-100 — except for NaCl, which shows no infiltration of either ion before doping. When more cations are inside the film, the negatively charged anions (in the solution) form an electric double layer at the interface with the polymer. Therefore, the electric field is directed into the electrolyte, causing a positive potential with the reference electrode, hence the positive $E_{Diffusion}$. Similarly, when the anion is preferentially more present in the film's surface (Na_2SO_3), the electric field is directed away from the electrolyte into the bulk, hence the negative $E_{Diffusion}$.

Discussion 4. Role of degree-of-dissociation and analysis of hydrated radius of ions

The degree of dissociation (α) of the Na^+ salts with ClO_4^-, Br^-, and Cl^- in water (at 25 °C with m=0.1mol/kg) is identical for the three electrolytes, with α = 0.857 ±0.001.[13-15] This suggests that the degree of dissociation of the organic salts' does not play a role in the differences observed in OECT performances. In contrast, the sulfite anion in $Na_2SO_{3\ (aq)}$ comes from a weak acid (sulfurous acid pK_a=6.91) $H_2SO_{3\ (aq)}$. Thus, it is expected that the Na_2SO_3 does not undergo complete dissociation in water, which contributes to the inferior performance of the OECT devices operating in $Na_2SO_{3\ (aq)}$.

Further, we calculated the hydration shells for the ions in each electrolyte through MD simulations (**Figures S9-S12**). A larger hydrated radius indicates a larger ion size, so water-ion interactions start at longer distances. The solvation shells of four NaX salts (X = Cl^-, Br^-, ClO_4^-, SO_3^{2-}) were calculated using the integrated radial distribution function at various distances, starting with a bound salt complex. For Na_2SO_3, a cubic box filled with 108 water molecules was used with two Na^+ cations and one SO_3^{2-} anion, while for all other salts, 109 water molecules were used along with one Na^+ and one X^- anion. Overall, the cubic box contains 111 molecules. For all cases, the corresponding concentration is 0.1 M.

At increasing timescale, the salts dissociate with increasing Na-X distance except for Na_2SO_3. The Na-Cl distance elongates from 2.36 Å to 6.94 Å at the end of the 50 ps, pointing out the preference for a dissociated state of NaCl. Similarly, Na-Br distance increases, but to a larger extent, becomes 18.42 Å. The Na-Cl distance in $NaClO_4$ becomes 13.80 Å. The Na-O (O atom in SO_3 group) distance in Na_2SO_3 (2.53 Å) reaches only 2.81 Å.

Looking closely at the hydration shell values, we find that NaCl and NaBr exhibit similar initial solvation structures. The hydration of Na^+ begins at 2.40 Å while it starts at 3.12 Å for Cl^- ion (**Figure S9**). In the case of NaBr, the hydration of Na^+ begins at 2.40 Å, like in NaCl, while Br^- hydration occurs at 3.28 Å (**Figure S10**). As the size of the ions increases, the first hydration occurs at more elongated distances. For example, Na^+ and ClO_4^- hydration begins at 2.96 and 3.64 Å, respectively (**Figure S11**). For the Na_2SO_3, Na^+ hydration is observed at 2.72 Å, whereas SO_3^{2-} hydration is observed at 3.49 Å (**Figure S12**). Although hydration shells look similar for $NaClO_4$ and Na_2SO_3 as polyatomic salts, they have different dissociation limits. At a distance of 3.7 Å, where all salts are dissociated and each ion is surrounded by a hydration shell, Cl^- and Br^- ions have five water molecules in their hydration shells, ClO_4^- has one, and SO_3^{2-} has three. In the case of Na^+ ions, the hydration shell consists of six water molecules in NaCl and NaBr, two in $NaClO_4$, and four in Na_2SO_3. Consequently, the increased relative mass uptake observed in $NaClO_4$ electrolyte is not due to the size of the hydration shell but rather to non-covalent interactions between the polymer side chains and the ClO_4^- ions.

In accordance with solvation shell analysis, $NaClO_4$ poses a slightly smaller hydration shell in comparison to NaBr, implying a less solvated anion nature. Therefore, the higher relative mass uptake of polymer with $NaClO_4$ cannot be explained by the size of solvation shell. To inspect the electrolyte-polymer interactions, we modeled two dimer models having NaBr and $NaClO_4$. Dimer-electrolyte interaction is slightly stronger by 1.6 kcal/mol in the presence of $NaClO_4$, likely due to a more stabilizing H-bond network. However, this slight difference is within the

error of DFT so we also tested tetramer models as a larger and computationally tractable model (**Figure 5b**). In the tetramer model, the interaction energy of $NaClO_4$ and tetramer hydrophilic side chains is noticeably more favored compared to NaBr by around 4.6 kcal/mol. This trend can explain the trapped ClO_4^- ions in the film, causing it to swell. We also calculated the interaction of NaCl with polymer in dimer and tetramer models. The predicted interaction energy is -1.8 kcal/mol in the tetramer model, comparable to the interaction energy of NaBr (-1.7 kcal/mol).

Next, we calculated the diffusion coefficients for all four salts in aqueous solution, as summarized in **Table S2**. Br^- displays the highest diffusivity, followed by Cl^- and ClO_4^-. ClO_4^- ions with a relatively low diffusion coefficient can be associated with the fact that Na^+ and ClO_4^- ions are trapped inside the polymer film. As for Na_2SO_3, having the smallest diffusion coefficient is consistent with its dissociation limit, preventing the transport of ions between the electrolyte and polymer.

Table S2. Calculated diffusion coefficients, D, in cm^2/s (X = Cl^-, Br^-, ClO_4^-, SO_3^{2-}).

Electrolyte	**D (Na^+)**	**D (X^-)**
NaCl	1.05E-5	3.58E-5
NaBr	9.65E-6	1.71E-5
$NaClO_4$	6.22E-6	1.61E-5
Na_2SO_3	6.46E-6	7.57E-6

Discussion 5. The stepwise formation of the radical anion

For polymers based on the alkyl-substituted NDI-T2 units, two prominent reduction peaks are attributed to the stepwise formation of a radical- then di-anion species.[16,17] Trefz et al. showed with in-situ conductance and CV measurements that the first reduction peak is assigned to forming the radical-anion species on the NDI monomer, and is directly related to the conductive property of the film when this redox polymer is formed.[16] On the other hand, the second reduction peak at higher potentials shows that the polymer in its di-anionic state loses most of its conductive properties. To prove that the two peaks that appear in the selected biasing window are associated with the stepwise formation of the radical anion and not the di-anion (0V to -0.6V vs. Ag/AgCl), we performed in-situ conductance (**Figure S14**) of P-100 films in NaCl, and $NaClO_4$. The conductance of the two doped polymers reaches maximum values around -0.6V *vs*. Ag/AgCl, suggesting no di-anion formation at these potentials. Importantly, in-situ conductance measurements show that when the first redox peak forms for the film operating in NaCl, we calculated a conductance 8 folds higher than the film operating in $NaClO_4$ at the same potential.

Moreover, the in-situ electrochemical UV-Vis spectroscopy (**Figure S15**) shows the absorption changes in response to applied doping potentials. The UV-Vis measurements show two prominent absorption bands, around 394 nm and 722 nm, attributed to the π-π* transition and intramolecular charge transfer (ICT), respectively.[18] The electrochemical doping of these films (0V to -0.6V vs. Ag/AgCl) in various electrolytes shows bleaching of the ICT signal and the emergence of an absorption structure at 513 nm attributed to forming the radical anion.[16] We do not observe bipolaron formation at this operation range.

Table S3. The calculated values of the half-wave potentials $E^{I}_{1/2}$ and $E^{II}_{1/2}$ and the peak-to-peak separation E^{I}_{pp} and E^{II}_{pp} extracted from the CV traces in **Figure S13**. The half-wave potentials are calculated according to the following equation[21] $E_{1/2} = (E_{p,red} + E_{p,ox})/2$

Electrolyte	$E^{I}_{1/2}$ (mV)	$E^{II}_{1/2}$ (mV)	E^{I}_{pp} (mV)	E^{II}_{pp}(mV)
NaBr	-363	-559	102	132
NaCl	-341	-543	122	160
$NaClO_4$	-405	-555	136	160
NaPSS	-403	-600	202	160
Na_2SO_3	-407	-549	136	146

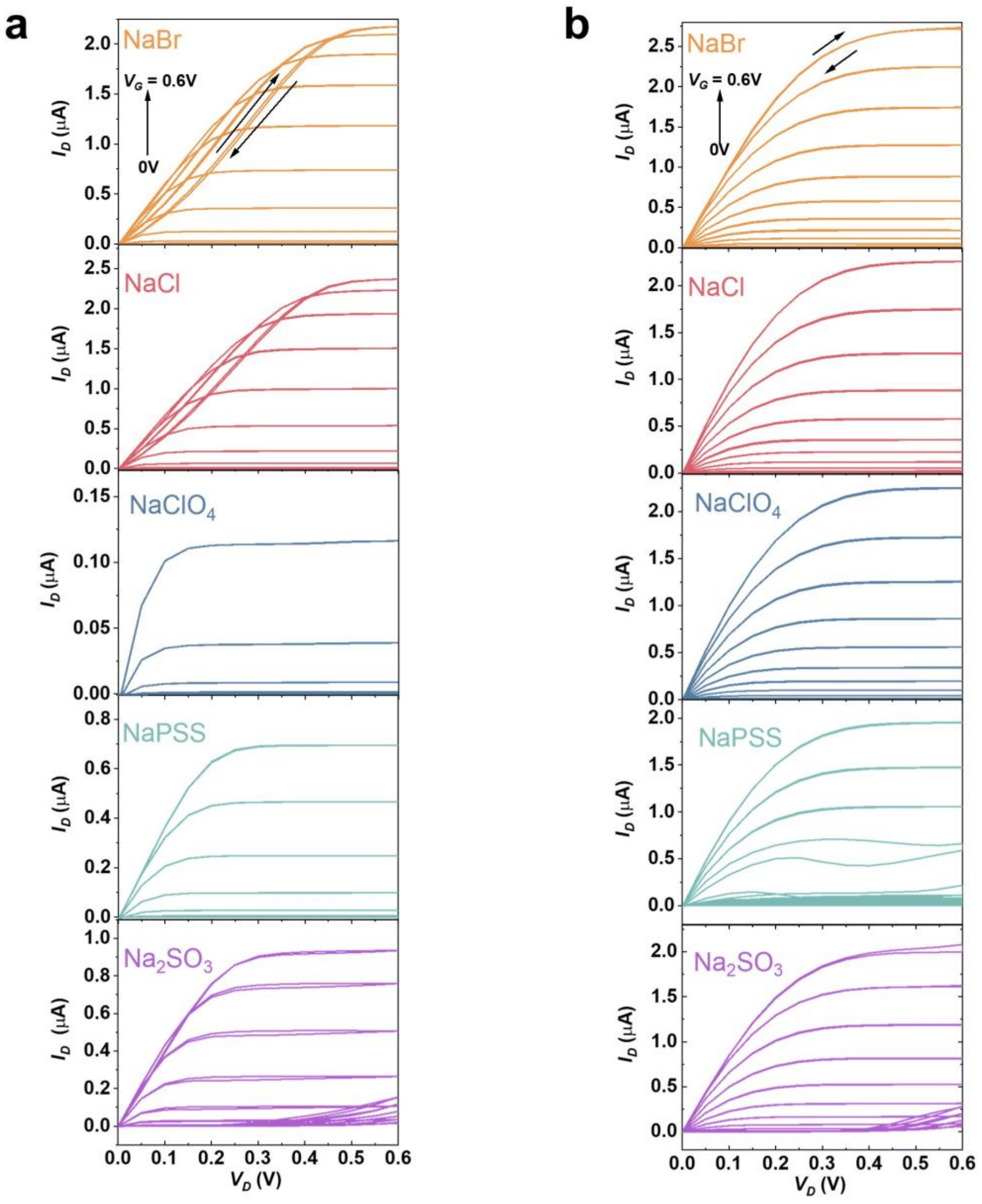

Figure S1. Output curves of **a)** P-100 and **b)** BBL devices in the different electrolytes tested (Na^+ concentration is maintained at 0.1M in all electrolytes). Arrows indicate the scan direction. The scan rate was 100 mV/s.

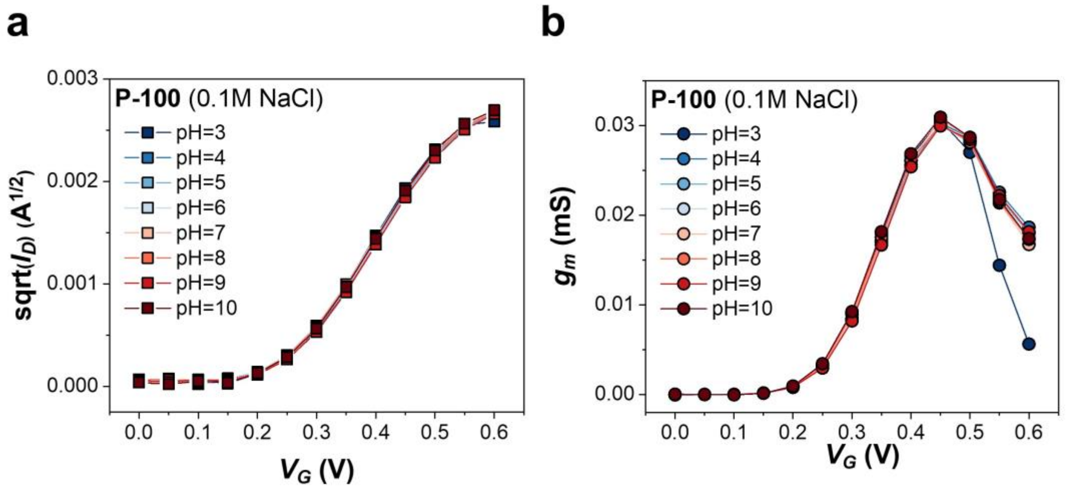

Figure S1. **a**) the square root of the I_D, and **b**) g_m as a function of V_G extracted at $V_D = 0.6$V for P-100 OECTs. The devices were measured in pH-controlled NaCl solutions($5 \leqslant pH \leqslant 9.8$). The V_{Th} is 0.22 V in all electrolytes. Transconductance behavior is identical at all pH values except at pH=3, where the g_m declines steeper with increasing bias.

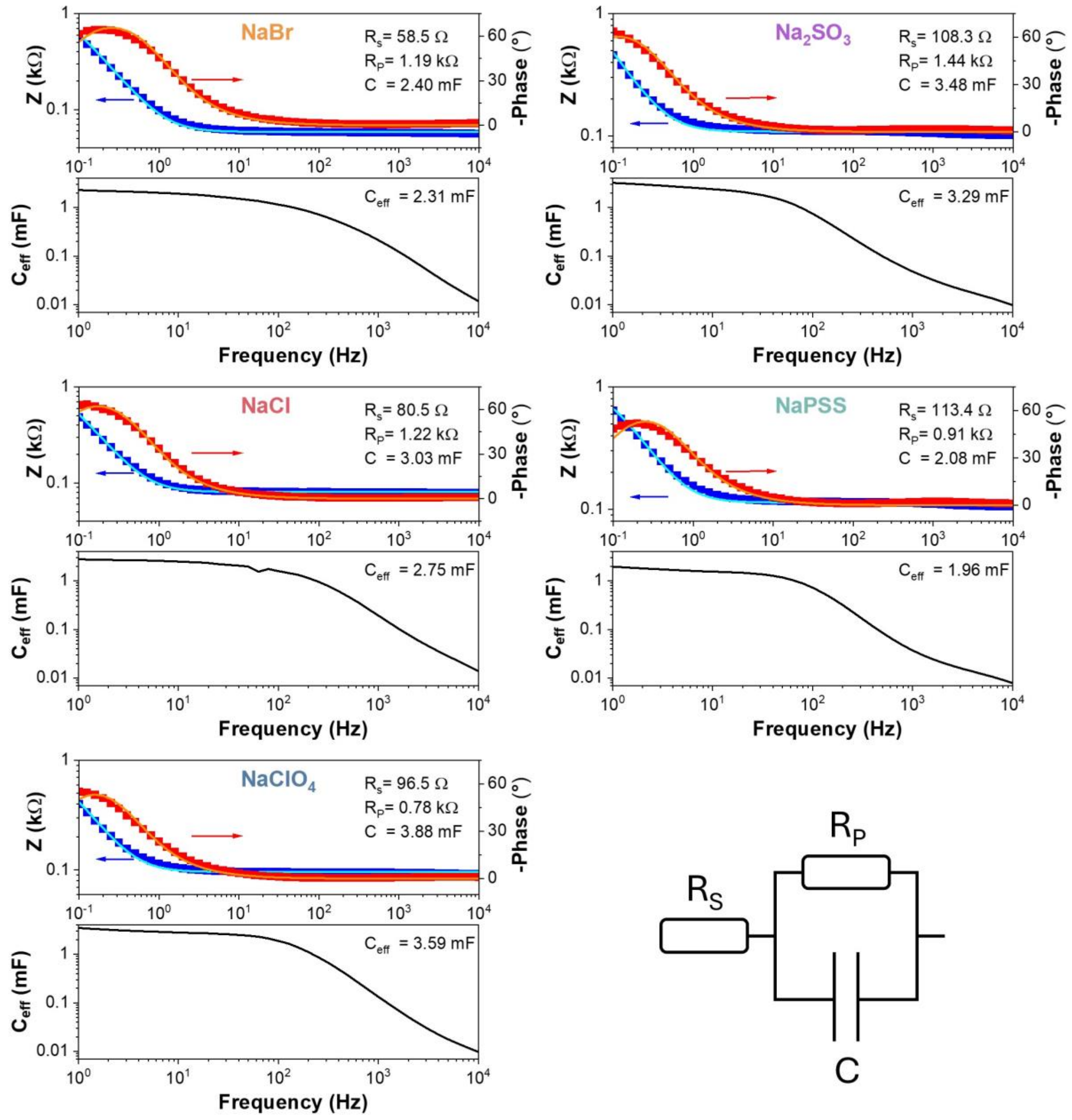

Figure S2. Bode plots for the P-100 films electrochemically doped in various electrolytes at an offset bias (V_{offset}) that yields $g_{m,max}$. The modulus of the impedance, |Z| (blue symbols), and phase (red symbols) are plotted as a function of frequency. EIS fits are shown as light-colored lines (|Z| fit, cyan; phase fit, orange). In addition to the capacitance values generated by the $R_s(R_p||C)$ equivalent circuit fits, below each Bode plot, we calculate the C_{eff} versus frequency The capacitance values generated by the $R_s(R_p||C)$ fit match those calculated from C_{eff} (at f =1 Hz).

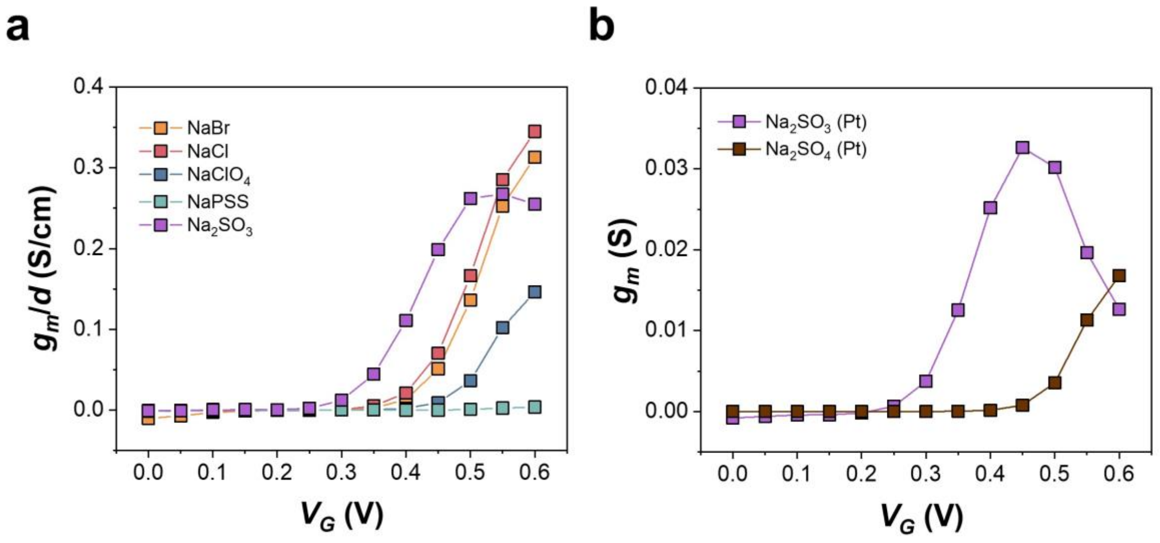

Figure S4. a) The transconductance measurements of P-100 OECTs operating in the various electrolytes using a polarizable Pt electrode. These measurements show a shift of V_{Th} to higher values and lower transconductance values except for OECTs operating in Na_2SO_3. V_D=0.6V; **b)** Transconductance measurements compare the effect of two electrolytes, sodium sulfite (Na_2SO_3) and sodium sulfate (Na_2SO_4). The electrolyte bearing the stable sulfate ion performs inferior to the other electrolytes. The improved performance of the channels in the electrolyte comprising the easily oxidizable sulfite ion is attributed to the faradaic reactions occurring at the Pt/SO_3^{2-} interface.

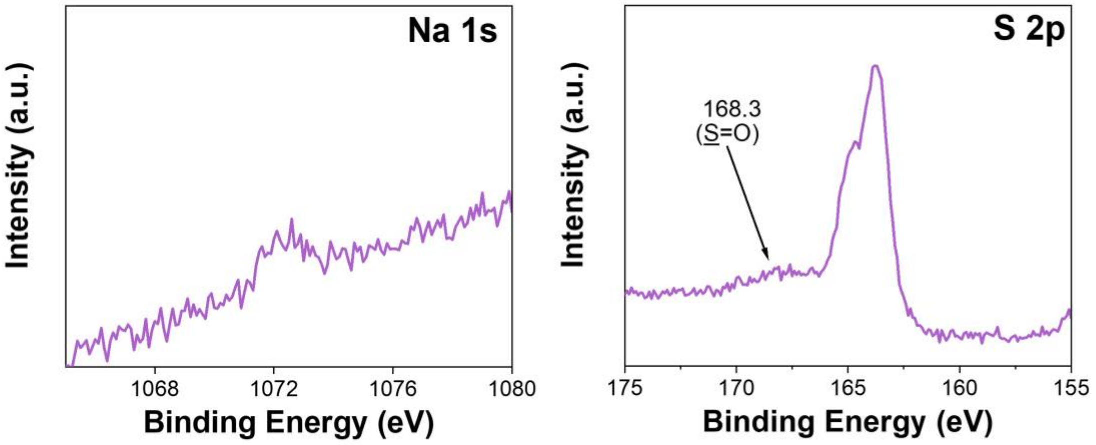

Figure S5. High-resolution XPS spectra showing (**a**) a small signal of Na^{+} peak in the film and **(b)** a slight bump at 163.3 eV attributed to the S=O bond in the SO_3^{2-} anion in the P-100 film immersed in the Na_2SO_3 electrolyte.

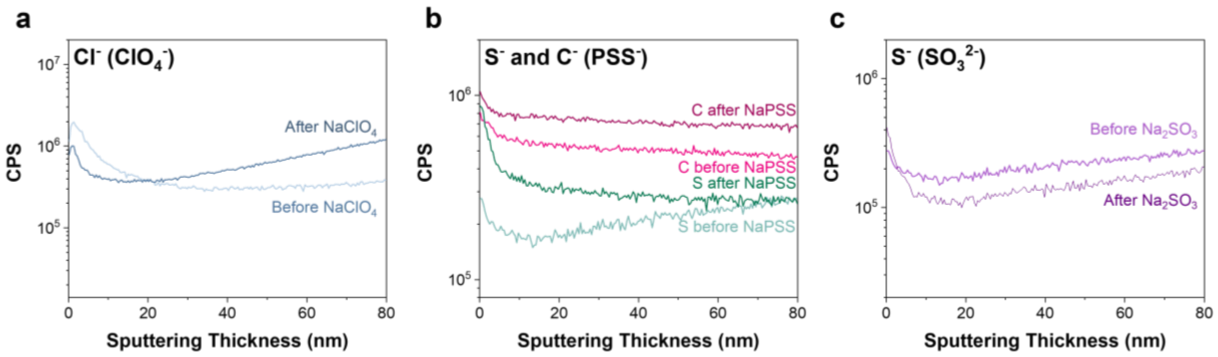

Figure S6. The SIMS depth-profiling curves of the anions of the three P-100 films before and after exposure to the electrolytes. **a**) Beyond 46 nm, the films show a higher signal of Cl^- after exposure to $NaClO_4$, indicating the presence of ClO_4^- throughout the film's bulk. **b**) the relatively similar levels of ionic C^- and S^- suggest that PSS^- do not infiltrate the film or that the NaPSS layer accumulates on top of the film within the etching depth. **c**) The S^- signal detected from the films exposed to Na_2SO_3 is of similar amplitude to that of the pristine films, suggesting no in-depth anion infiltration.

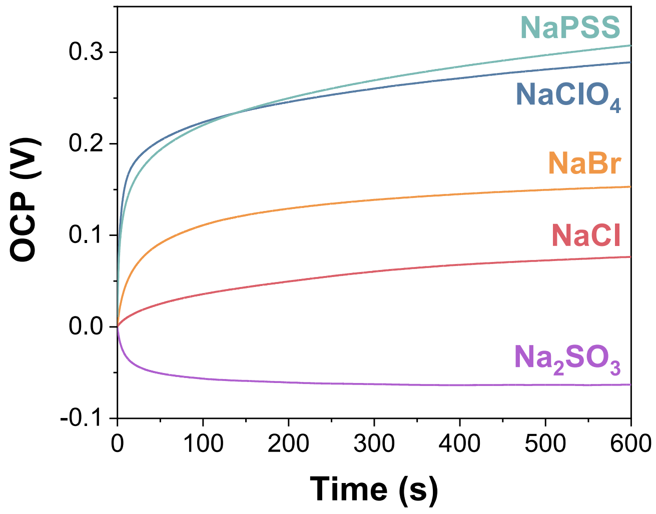

Figure S7. The open-circuit potential (OCP) of P-100 films when exposed to the five electrolytes.

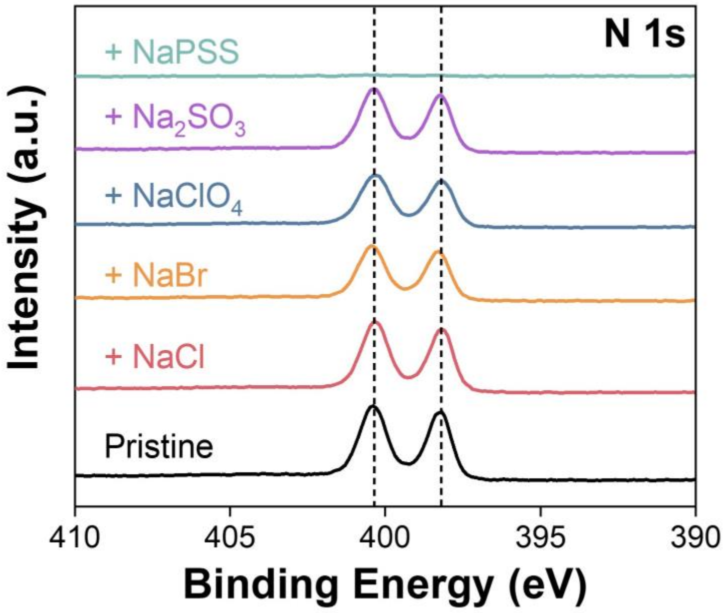

Figure S8. High-resolution XPS spectra of the BBL films before and after exposure to different electrolytes. N 1s signal is visible for all electrolytes without a shift, indicating that the BBL backbone is chemically intact. NaPSS represents a unique case, where the disappearance of the BBL N 1s signal is associated with the formation of a NaPSS layer on top of the film, as observed for P-100.

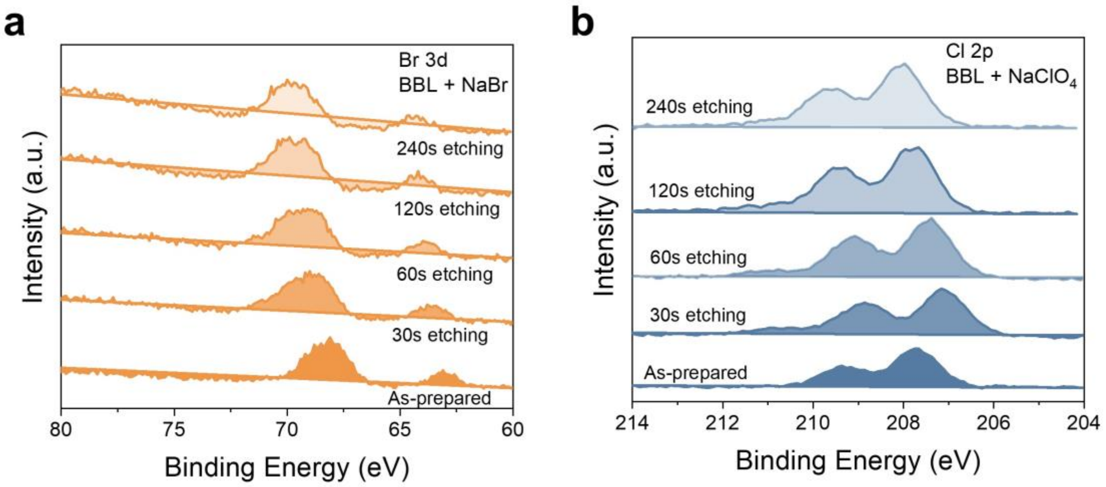

Figure S9. High-resolution XPS spectra of BBL films before and after exposure to (**a**) NaBr and (**b**) $NaClO_4$. The Br 3d peaks (**a**) and Cl 2p peaks (**b**) are displayed as a function of etching time, revealing the presence of the respective anions at various depths within the BBL films.

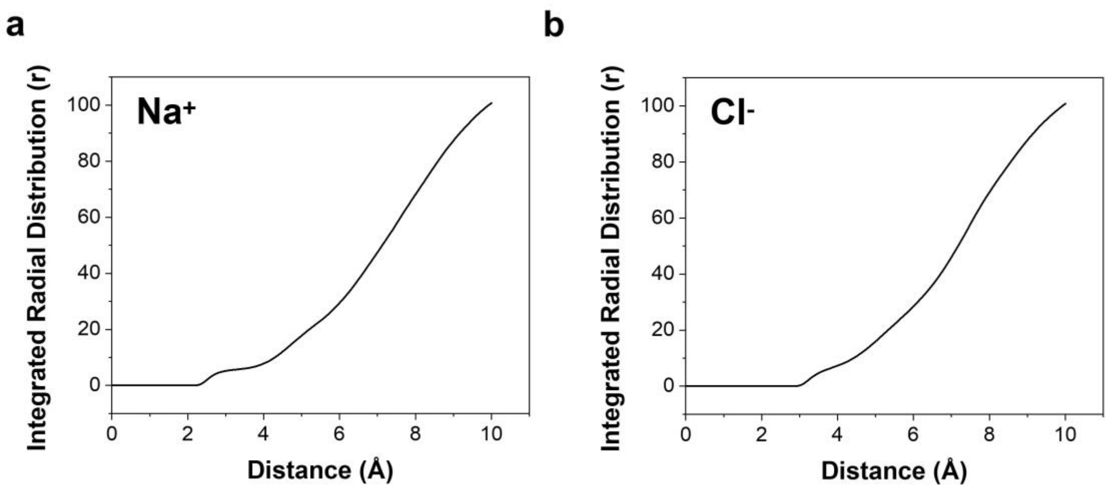

Figure S10. NaCl hydration shell for 0.1 M NaCl in aqueous solution. Hydration shell calculation for **a**) Na^+ and **b**) Cl^-. Distance represents the distance of each ion from one another. As the experiment progresses, the distance between Na^+ and Cl^- increases with each ion becoming larger with hydration.

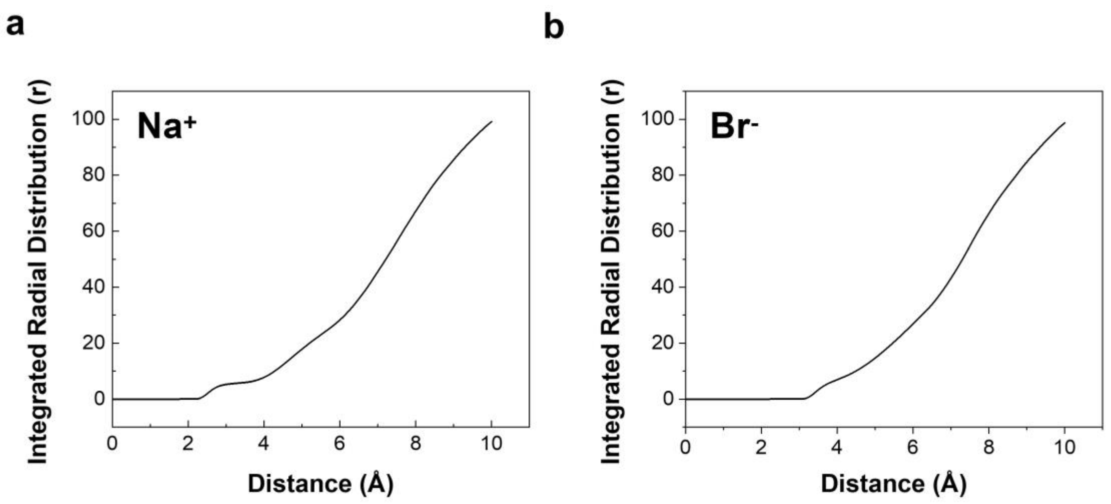

Figure S11. NaBr hydration shell for 0.1 M NaBr in aqueous solution. Hydration shell calculation for **a**) Na^+ and **b**) Br^-.

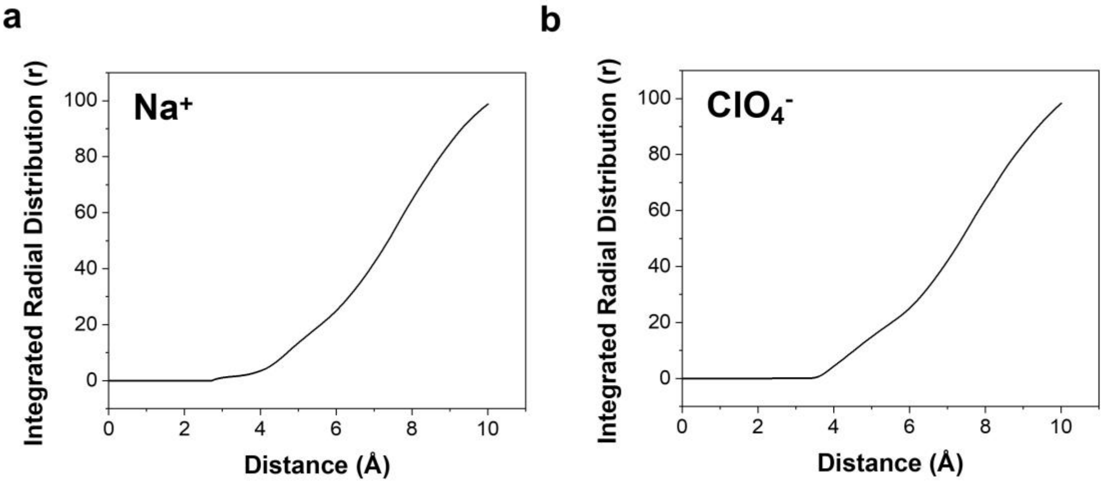

Figure S12. $NaClO_4$ hydration shell for 0.1 M $NaClO_4$ in aqueous solution. Hydration shell calculation for **a**) Na^+ and **b**) ClO_4^-.

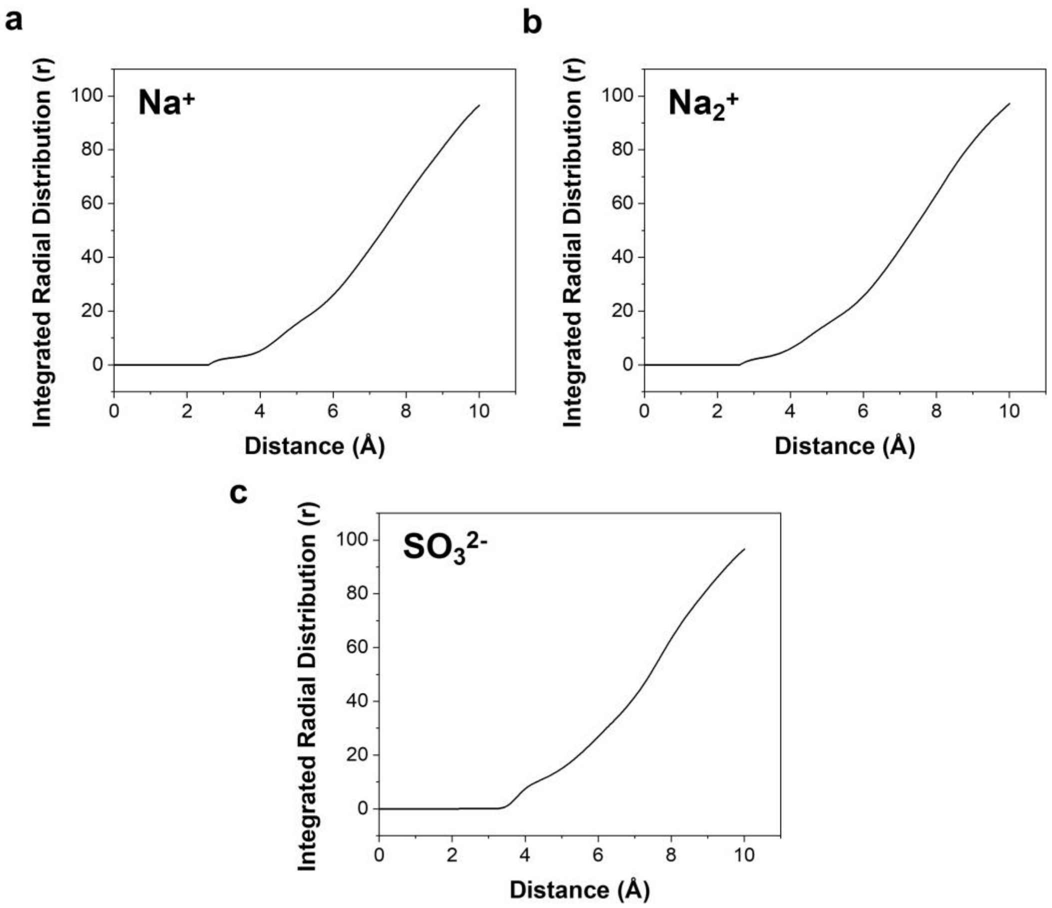

Figure S13. Na_2SO_3 hydration shell for 0.1 M Na_2SO_3 in an aqueous solution. Hydration shell calculation for **a**) Na^+, **b**) Na_2^+, and **c**) SO_3^{2-}.

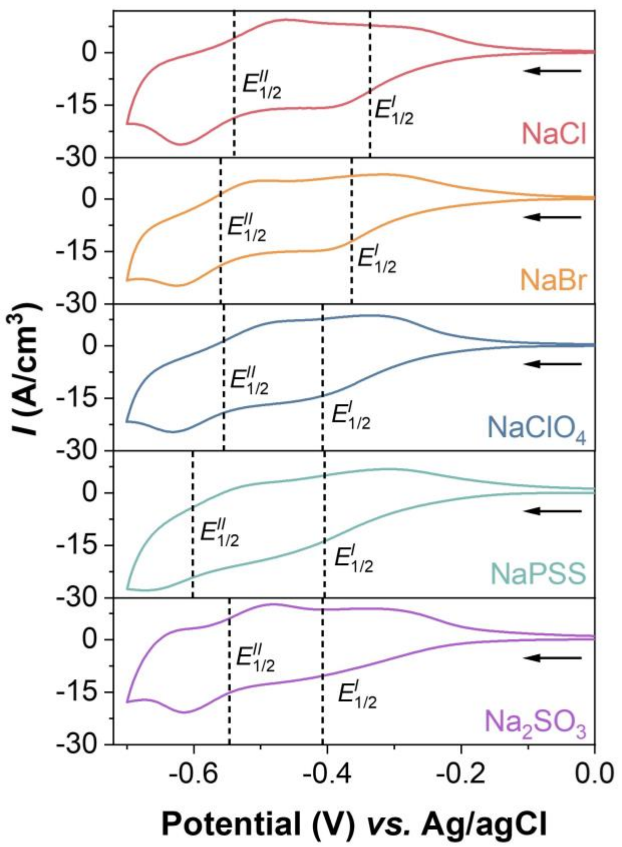

Figure S14. Cyclic voltammograms of P-100 in the different electrolytes studied. The scan rate is 80 mV/s, and the arrow indicates the scan direction.

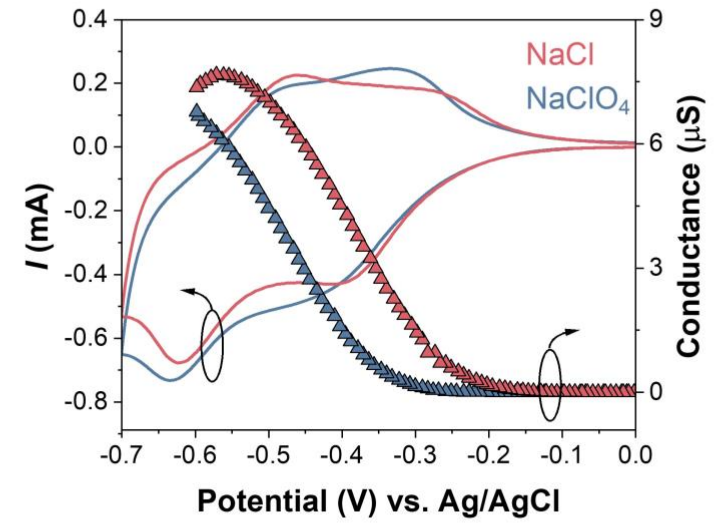

Figure S15. In-situ conductance measurements showing CV and conductance plots for P-100 films operating in NaCl (0.1M) and $NaClO_4$ (0.1M). The conductance increases as the second reduction peak forms, verifying the formation of a radical anion, not a dianion.

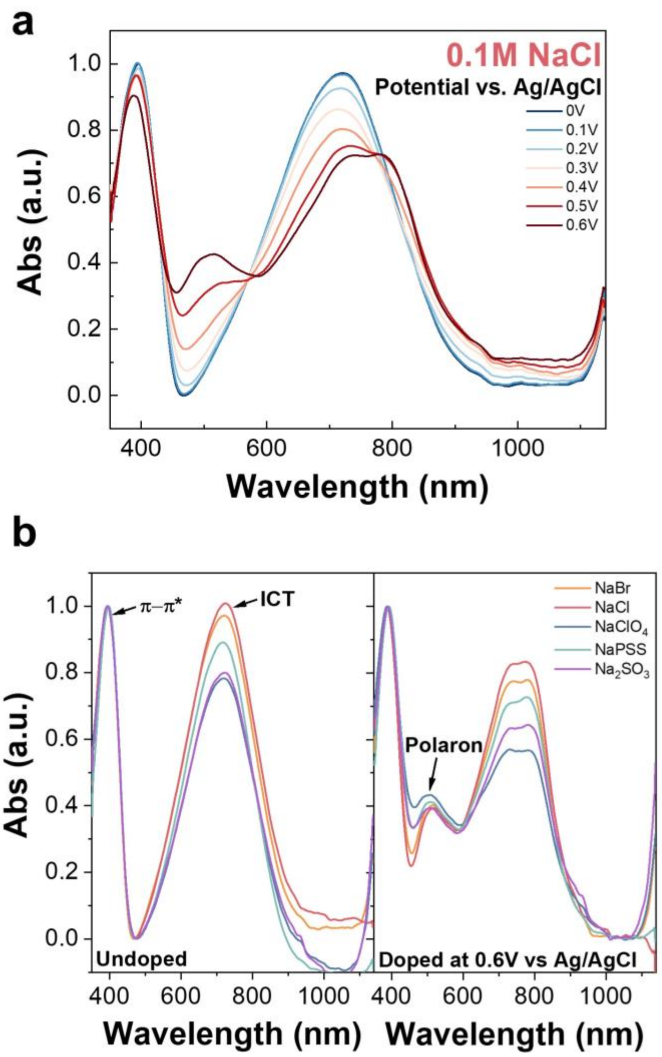

Figure S16. In situ UV-VIS-NIR spectra of P-100 films doped in **a**) 0.1 M NaCl from 0 V to 0.6 V vs. Ag/AgCl and **b**) all electrolytes in the undoped and the doped state (at 0.6V vs. Ag/AgCl). All doped films show similar absorption spectra before and after doping.

References

1 Savva, A. *et al.* Influence of Water on the Performance of Organic Electrochemical Transistors. *Chemistry of Materials* **31**, 927-937 (2019). https://doi.org/10.1021/acs.chemmater.8b04335

2 Novotny, P. & Sohnel, O. Densities of binary aqueous solutions of 306 inorganic substances. *Journal of Chemical & Engineering Data* **33**, 49-55 (1988). https://doi.org/10.1021/je00051a018

3 Okur, H. I. *et al.* Beyond the Hofmeister Series: Ion-Specific Effects on Proteins and Their Biological Functions. *The Journal of Physical Chemistry B* **121**, 1997-2014 (2017). https://doi.org/10.1021/acs.jpcb.6b10797

4 Collins, K. D. Ions from the Hofmeister series and osmolytes: effects on proteins in solution and in the crystallization process. *Methods* **34**, 300-311 (2004). https://doi.org/https://doi.org/10.1016/j.ymeth.2004.03.021

5 Salis, A. & Ninham, B. W. Models and mechanisms of Hofmeister effects in electrolyte solutions, and colloid and protein systems revisited. *Chemical Society Reviews* **43**, 7358-7377 (2014). https://doi.org/10.1039/C4CS00144C

6 Moghaddam, S. Z. & Thormann, E. The Hofmeister series: Specific ion effects in aqueous polymer solutions. *Journal of Colloid and Interface Science* **555**, 615-635 (2019). https://doi.org/https://doi.org/10.1016/j.jcis.2019.07.067

7 dos Santos, A. P., Diehl, A. & Levin, Y. Surface Tensions, Surface Potentials, and the Hofmeister Series of Electrolyte Solutions. *Langmuir* **26**, 10778-10783 (2010). https://doi.org/10.1021/la100604k

8 Nishida, K. *et al.* Salting-out and salting-in effects of amphiphilic salt on cloud point of aqueous methylcellulose. *Process Biochemistry* **59**, 52-57 (2017).

9 Skavås, E. & Hemmingsen, T. Kinetics and mechanism of sulphite oxidation on a rotating platinum disc electrode in an alkaline solution. *Electrochimica Acta* **52**, 3510-3517 (2007). https://doi.org/https://doi.org/10.1016/j.electacta.2006.10.038

10 Enache, A.-F., Dan, M. L. & Vaszilcsin, N. Electrochemical Oxidation of Sulphite in Neutral Media on Platinum Anode. *International Journal of Electrochemical Science* **13**, 4466-4478 (2018). https://doi.org/https://doi.org/10.20964/2018.05.07

11 Xin, W. *et al.* High-performance silk-based hybrid membranes employed for osmotic energy conversion. *Nature Communications* **10**, 3876 (2019). https://doi.org/10.1038/s41467-019-11792-8

12 Chen, W. *et al.* Improved Ion Transport in Hydrogel-Based Nanofluidics for Osmotic Energy Conversion. *ACS Central Science* **6**, 2097-2104 (2020). https://doi.org/10.1021/acscentsci.0c01054

13 Heyrovská, R. Ionic Concentrations and Hydration Numbers of "Supporting Electrolytes". *Electroanalysis* **18**, 351-361 (2006). https://doi.org/https://doi.org/10.1002/elan.200503416

14 Heyrovska, R. Unified Thermodynamics for All Concentrations of Electrolytes Based on Hydration and Partial Dissociation (Without Activity Coefficients, 1995-). *ECS*

Meeting Abstracts **MA2016-01**, 1642 (2016). https://doi.org/10.1149/MA2016-01/34/1642

15 Heyrovská, R. Degrees of dissociation and hydration numbers of alkali halides in aqueous solutions at 25° C (some up to saturation). *Croatica chemica acta* **70**, 39-54 (1997).

16 Trefz, D. *et al.* Electrochemical Investigations of the N-Type Semiconducting Polymer P(NDI2OD-T2) and Its Monomer: New Insights in the Reduction Behavior. *The Journal of Physical Chemistry C* **119**, 22760-22771 (2015). https://doi.org/10.1021/acs.jpcc.5b05756

17 Gross, Y. M. *et al.* From Isotropic to Anisotropic Conductivities in P(NDI2OD-T2) by (Electro-)Chemical Doping Strategies. *Chemistry of Materials* **31**, 3542-3555 (2019). https://doi.org/10.1021/acs.chemmater.9b00977

18 Liu, J. *et al.* Enhancing Molecular n-Type Doping of Donor–Acceptor Copolymers by Tailoring Side Chains. *Advanced Materials* **30**, 1704630 (2018). https://doi.org/https://doi.org/10.1002/adma.201704630
